# Microenvironmental Behaviour of Nanotheranostic Systems for Controlled Oxidative Stress and Cancer Treatment

**DOI:** 10.3390/nano12142462

**Published:** 2022-07-18

**Authors:** Yaser Rehman, Hamzeh Qutaish, Jung Ho Kim, Xu-Feng Huang, Sadia Alvi, Konstantin Konstantinov

**Affiliations:** 1 Institute for Superconducting and Electronics Materials (ISEM), University of Wollongong (UOW), Wollongong, NSW 2522, Australia; yr997@uowmail.edu.au (Y.R.); hqmq581@uowmail.edu.au (H.Q.); jhk@uow.edu.au (J.H.K.); 2 Illawarra Health & Medical Research Institute (IHMRI), University of Wollongong (UOW), Wollongong, NSW 2522, Australia; xhuang@uow.edu.au; 3 Drug Discovery Biology, Monash Institute of Pharmaceutical Sciences, Monash University, Parkville, VIC 3052, Australia; sadia.alvi@monash.com.au

**Keywords:** ROS, nanotheranostics, oxidative stress, apoptosis, oxide nanoparticles, cellular internalisation, cancer

## Abstract

The development of smart, efficient and multifunctional material systems for diseases treatment are imperative to meet current and future health challenges. Nanomaterials with theranostic properties have offered a cost effective and efficient solution for disease treatment, particularly, metal/oxide based nanotheranostic systems already offering therapeutic and imaging capabilities for cancer treatment. Nanoparticles can selectively generate/scavenge ROS through intrinsic or external stimuli to augment/diminish oxidative stress. An efficient treatment requires higher oxidative stress/toxicity in malignant disease, with a minimal level in surrounding normal cells. The size, shape and surface properties of nanoparticles are critical parameters for achieving a theranostic function in the microenvironment. In the last decade, different strategies for the synthesis of biocompatible theranostic nanostructures have been introduced. The exhibition of therapeutics properties such as selective reactive oxygen species (ROS) scavenging, hyperthermia, antibacterial, antiviral, and imaging capabilities such as MRI, CT and fluorescence activity have been reported in a variety of developed nanosystems to combat cancer, neurodegenerative and emerging infectious diseases. In this review article, theranostic in vitro behaviour in relation to the size, shape and synthesis methods of widely researched and developed nanosystems (Au, Ag, MnO*_x_*, iron oxide, maghemite quantum flakes, La_2_O_3−*x*_, TaO*_x_*, cerium nanodots, ITO, MgO_1−*x*_) are presented. In particular, ROS-based properties of the nanostructures in the microenvironment for cancer therapy are discussed. The provided overview of the biological behaviour of reported metal-based nanostructures will help to conceptualise novel designs and synthesis strategies for the development of advanced nanotheranostic systems.

## 1. Introduction

Nanoparticles (NPs) offer a unique combination of physiochemical properties for the treatment of various disease. Their multifunctional use in therapeutics, imaging, drug delivery and diagnostics is increasing day by day [1,2]. In the last decade, oxide-based nanotheranostic systems have rapidly expanded and emerged as a leading research field. The integration of therapeutic and imaging capabilities into a single nano-entity can form theranostic nanostructures [3]. An efficient theranostic NPs drug system must have requisite biocompatibility, biodegradability and adequate clearance. Other properties, such as tumour accumulation, drug delivery, immune system escape and selective targeting are also matters of interest in disease therapy and diagnostics [4]. In view of the theraputic aspects, NPs have excellently enhanced the efficiency of many cancer treatment methods, such as chemotherapy, radiation therapy, immunotherapy, targeted drug therapy and magnetic hyperthermia. Many in vitro and in vivo studies found an increase in reactive oxygen species (ROS) in most cancer treatment cases. New treatment strategies based on the augmentation of ROS levels to induce higher oxidative stress in cancer cells are being developed. Increased oxidative stress overwhelms the redox adaptation of cells, which is incompatible with the survival of cellular life and can eradicate tumour cells. ROS levels play a critical role in the development and progression of cancer, and are also considered to be vital for cancer treatment [5].

ROS are produced from exogenous as well as endogenous sources by enzymatic or non-enzymatic reactions. Exogenous sources include smoking, certain drugs, pollutants and alcohol, whereas infection, stress, ischemia, immune cell activation and mitochondrion reactions are sources of endogenous ROS. The enzymatic reactions for ROS production include phagocytosis, cytochromes reactions, prostaglandin and the respiratory chain. Non-enzymatic free radicals are generated by the reaction of oxygen with organic compounds when cells are exposed to radiation. Non-enzymatic ROS can also be generated by mitochondrion respiration.

ROS accumulation affects the physiological signalling network to initiate the pathological conversion of normal cells into malignant cells and contributes towards malignant cell proliferation. Excessive ROS generation may have a damaging effect on cell organelles, including cell membranes, lipids, proteins and DNA, and can cause cell death. Therefore, the scavenging of ROS to prevent oxidative stress or generation of more ROS to kill the cancer cells by elevating oxidative stress levels are promising strategies for cancer treatment.

The neutralisation of excess ROS is achieved through well-known enzymes, including glutathione; flavonoids; vitamins A, C and E or through antioxidant compounds that specifically scavenge ROS. Superoxide dismutase (SOD) is a metalloenzyme that exists abundantly in eukaryotes and prokaryotes utilise metal ions, such as manganese (Mn^2+^), iron (Fe^2+^), zinc (Zn^2+^) and copper (Cu^2+^) for the dismutation of the superoxide anion (O_2_^•−^) to oxygen (O_2_) and hydrogen peroxide (H_2_O_2_) [6]. The catalase (CAT) enzyme is involved in the decomposition of H_2_O_2_ into water (H_2_O) and O_2_ [7,8]. Similarly, many nanostructures or composite nanostructures based on Au, Ag, Mn, Mg Ce, Se, Fe, Ti, Zn, In, Bi, Ta, redox polymer and polyphenols are selectively employed in ROS scavenging or generation.

Various oxide NPs based non-invasive techniques, such as magnetic resonance imaging, optical imaging, computer tomography (CT), positron emission tomography, ultrasonic imaging and single photon emission computer tomography are being employed in disease treatment [9,10,11]. Nanomaterial-based radiolabels find applications in nuclear medicine imaging [12]. Silica-coated gold (Au) NPs are used as photoacoustic contrast agents for the imaging of sentinel lymph node. Supermagnetic theranostic NPs have been extensively studied for MRI and the local hyperthermia of tumour [1,13].

Although nanotheranostics have great potential in the field of nanomedicine, the generation of unwanted ROS and oxidative stress in normal cells and retention inside the body present a hurdle in the translation to effective and viable treatment strategies [14,15].

NP properties alter with the change in surface chemistry, size and shape, which ultimately depends upon synthesis conditions. The variations in pH, ROS level and other cellular and extracellular environments affect the biocompatibility and therapeutic and imaging properties of nanostructures. NPs can be specifically designed to optimise the desired performance under selective conditions. Furthermore, surface coating or encrustation and the formation of a composite or core shell structure have been adopted to increase the efficiency and biocompatibility of the nanostructures or nanomedicines. Combining the diagnostic and therapeutic properties into a single nano/nanocomposite entity presents great potential in the field of medicine.

**Figure 1:**
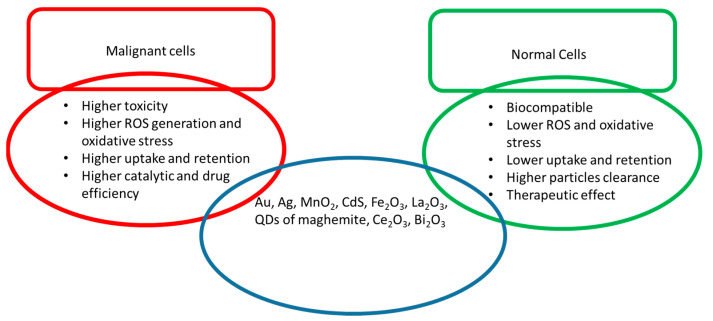
Functional properties of different metal and metal oxide NPs in malignant and normal cells.

There are a variety of conventional synthesis techniques, such as precipitation, gas condensation, sol–gel, laser ablation, sono-chemical, hydrothermal, spark discharge and microwave, which have been employed for the synthesis of the nanostructures of different metal oxides [16]. The modifications in synthesis techniques or strategies, the use of non-toxic chemical reagents and controlled processing parameters can enhance biocompatibility and increase surface therapeutic efficiency and imaging capabilities. Surface property modifications enhance the chemical interaction with a site-specific target, such as in the case of tumour cells [17,18]. In addition, they can be efficient carriers of drugs for the selective treatment of disease without harming the normal neighbouring tissues or cells. Enhanced permeability (EPR) and retention in the malignant tumour are also desired for efficient drug responses. In the case of NP-based immunotherapy, the system is designed for the controlled drug release, generation or scavenging of ROS in the complex tumour microenvironment by exploiting their enzymatic, pH, hypoxia, ultrasound, electricity and light-dependent response [19,20,21]. Nanoparticles’ internalisation or delivery to specific sites is usually obtained by passive and active targeting. Passive targeting involves the passage of nanoparticles through the leaky vasculature and accumulation within a tumour. In active targeting, molecules/ligands are attached to the NP’s surface for specific receptor acceptance [22]. Antioxidants, SOD conjugated polymers or metal-based NPs are employed as active and passive targeting simultaneously for intercellular and extracellular ROS management [8,23,24]. Cell culture studies can provide detailed information about the biological process at the basic level in an organism. The cell culture models are very useful in evaluating the toxicology and physiology of NPs/drugs, Figure 1. They play an important role in the development of vaccines, drugs, bioactive substances, diagnostic techniques, theranostic agents, food ingredients and cosmetics [25]. This review mainly focuses on the passive activity of NPs in disease (malignant) conditions with reference to normal cellular behaviour to record their effectiveness.

In the present article, the in vitro biological effects of nanotheranostic structures, such as (Au), silver (Ag), manganese (Mn)-based NPs, iron oxide nanostructures, oxygen-deficient La_2_O_3_, nanodots of cerium oxide, surface-encrusted nanostructures, maghemite quantum flakes, indium tin oxide (ITO), tantalum oxide (Ta_2_O_3_) and magnesium oxide (MgO) are summarised. The morphology and synthesis methods of nanostructures are also outlined. In particular, the biocompatible nature in relation to synthesis strategy is detailed in the current review. They show promising results in the treatment of various types of cancers and other emerging diseases. Advanced nano-oxide structures will shift the future of medicine to more compact, safe and economical therapeutic and diagnostic systems. The present review can provide a platform for the selection and design of an efficient nanomedicine or materials system to meet the challenges of current and emerging diseases.

## 2. Au-Based Nanostructures

Recently, Au NPs have received considerable interest in the field of medicine due to their theranaostics properties. The high specific surface area of NPs and excellent surface-dependent catalytic properties potentiate their use in a variety of biomedical and engineering applications. Au NPs are being used in solar cells, flash memory storage, pollution control, water and hydrogen purification and the catalytic oxidation of carbon monoxide. In the biomedical field, Au NPs are employed in genomics, cancer therapy, biosensors, drug delivery and cell monitoring and imaging [26,27,28,29,30]. 

Recent studies have shown that Au NPs have anti-tumour properties against breast, colon, lungs and liver cancers [31,32]. In addition to anti-tumour properties, the NPs’ effects on normal cells have also been studied. Similarly, Au NPs are also linked to cellular apoptosis through the generation of oxidative stress. J. Li et al. reported the induction of oxidative stress in Au NP-treated human lung fibroblast cells. The treatment with Au NPs caused lipid peroxidation, the upregulation of antioxidants, protein expression and stress-response genes [33]. The chemically synthesised Au NPs exhibited higher toxicity in comparison to biosynthesised and surface-coated/core shell NPs [31,34]. Fewer studies have reported that Au NPs can be synthesised for selective oxidative stress/toxicity in cancer cells and normal cells [35,36]. The size, shape, surface charge and surface treatment of NPs influence their circulation, retention and toxicity in the body. The extent of toxicity of Au NPs also depends on the method of production and functionalisation of NPs with polymeric substances. Bare Au NPs usually exhibit greater toxicity than functionalised and biogenic NPs. The size, morphology, synthesis methods and in vitro cellular effects of Au NPs on various cell lines are shown in Table 1. 

**Figure 2:**
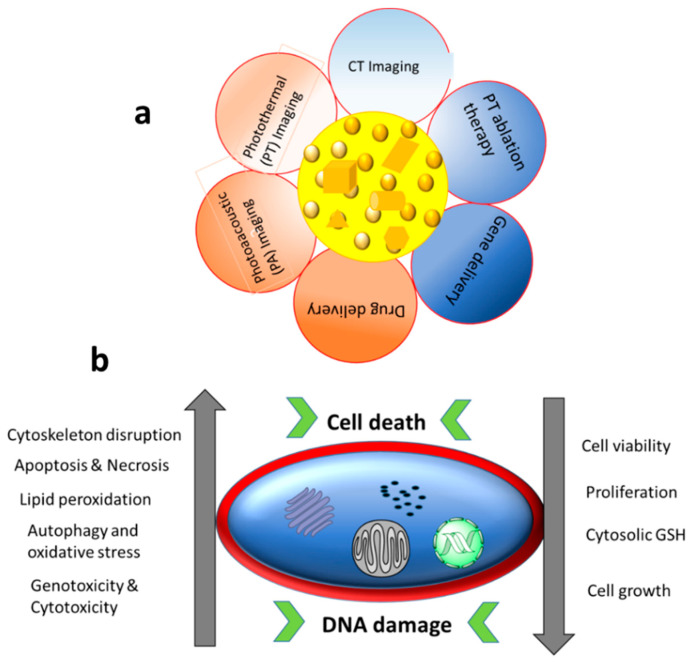
(**a**) Biological applications of Au NPs and (**b**) different contributing factors (with increasing or decreasing trends) towards DNA damage and cell death caused by treatment with Au NPs.

Au NPs absorb visible light and emit energy of a specific wavelength, which is used for diagnostic and light-mediated clinical treatments [37]. Due to the unique surface plasmonic properties of Au NPs, they are extensively employed in optical imaging. They offer an excellent choice for cellular visualisation as the scattering signal from Au NPs is much stronger than the background scattering from cellular components and tissues. Even the interaction of a single Au NP with biological systems can be visualised using dark field (DF) and bright field (BF) microscopies, differential interference contrast (DIC) microscopy and photothermal and photoluminescence methods [38]. Au NPs can offer multifunctional and theranostic treatment strategies with requisite biocompatibility, if developed by a suitable processing technique or with surface treatment using biocompatible materials. In recent studies, Au NPs expressed good biocompatibility, ease of synthesis, surface property modification, surface plasmonic properties and passive targeting for cancerous cells [22]. Au NPs are considered as a better choice for passive targeting due to their excellent EPR effect. Recently, Au NPs systems have been developed for the active targeting of cancerous cells. Various molecular attachments based on proteins, peptides, polymers, carbohydrates and antibodies are attached to the surface of Au NPs for specific receptor targeting [22,39]. The major applications and common cellular effects of Au NPs are shown in Figure 2.

**Figure 3:**
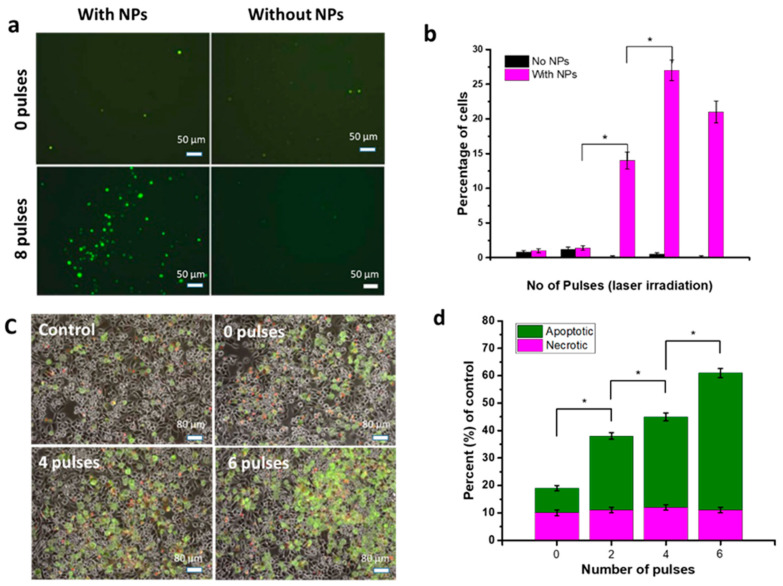
(**a**) Fluorescence images of ROS generation in Burkitt lymphoma cells after laser irradiation in the presence and absence of NPs. (**b**) Percentage of cells with respect to ROS presence. Where * *p* ˂ 0.0001. (**c**) Fluorescence distribution, red (necrosis) and green (apoptosis) colours depicting the death of epithelial breast cancer cells due to ROS production by laser irradiation and (**d**) bar chart showing the percentage of necrotic (red) and apoptotic (green) cells. Where * *p* ˂ 0.0003. Images and graphs are reproduced with permission from [40], copyright 2013, Springer Nature, and representing the overall trend of results.

Minai et al. reported the increased generation of ROS during exposure to laser pulses in the presence of Au NPs [40]. After irradiation (8 pulses) in the presence of Au NPs, Burkitt lymphoma cells showed an increased amount of ROS (green dots, Figure 3a) in comparison to the control, as observed by fluorescence microscopy. A relative percentage increase in ROS with and without NPs and laser irradiations is shown in Figure 3b [40]. Similarly, fluorescence imaging on NP-treated epithelial breast cancer cells showed increased green fluorescence after laser pulse exposure (Figure 3c). In the case of 06 pulses of laser irradiation and NP treatment of epithelial breast cancer cells, about a 55% increase in apoptotic cells resulted, compared to the control cells (Figure 3d) [40].

**Table 1:** Cellular responses of different cell culture models with reference to size, shape and synthesis of Au NPs.

Cell Line	NP Shape and Size	Synthesis Method	Effect
Human breast cancer cells (MDA-MB 231)	Spherical (25–30 nm)	Chemical reduction	Decrease in proliferation [41], caspase-3 activation and DNA fragmentation [42,43].
Human keratinocyte cell line (HaCaT)	Nanorods (30 nm)	Citrate reduction	Au NPs caused necrosis and apoptosis, leading to cell death [44,45].
Human liver cell line (HL7702 cells)	Spherical (5–100 nm)	Citrate reduction	Decrease in cytosolic GSH, depolarisation of transmembrane potential in the mitochondria, followed by apoptosis and death [46].
Human lung adenocarcinoma (A549) cells	Spherical (30 nm)	Citrate reduction	Upregulation of apoptotic genes (including bax, bak and caspase-3), induction of apoptosis through depletion of ATP [47].
Human leukaemia (HL-60)	Spherical (30, 50, 90 nm)	Commercial (CymitQuı’mica)	Membrane damage, reduction in GSH and generation of ROS, increase in cell mortality [48].
MRC human foetal lung fibroblast cell	Unknown shape (20 nm)	Citrate reduction	Increase in oxidative stress, downregulation of cell cycle gene, inhibition of cell proliferation and DNA damage [27,49].
Human foetal osteoblast (hFOB 1.19)	Spherical (38–60 nm)	Citrate-based reduction	Cell membrane penetration, ultrastructural changes and cell death [50].
Human osteosarcoma cells (MG63)	Spherical (38–60 nm, 6.3 nm), nanorods (18, 39 nm), stars (215 nm)	Citrate-based reduction, chemical reduction-based immobilisation method	Increase in reactive oxygen species, disruption of membrane potential of mitochondrion, apoptosis and cell death [27,50,51].
Human osteosarcoma cells 143B	Spherical (6.3 nm), nanorods (18, 39 nm), stars (215 nm)	Chemical reduction-based immobilisation method	Nanostars caused higher cytotoxicity, apoptosis and cell death than nanorods and nanospheres [51,52].
Human colorectal adenocarcinoma cells (HT29)	Unknown shape (15 nm)	Institute of biochemistry & physiology of plants & microorganisms, Russian academy of sciences.	Cytotoxic and anti-cohesive effects, multicellular spheroid formation, reduced proliferation, apoptosis and necrosis [31,53].
Human A549 cell line	Unknown (39–45 nm)	Commercial (Sigma-Aldrich, St. Louis, MO, USA	Growth inhibition, apoptosis and autophagy, DNA damage [54].
Human HepG2 cells	Spherical (20 nm)	Citrate reduction	Size-dependent toxicity, comet tail intensity and tail moment were observed [55,56].
Human colorectal adenocarcinoma cells (HT29)	Unknown shape (50–100 nm)	Photonic Technology (IPHT Jena, Germany)	Inhibition of cell proliferation and angiogenesis, reduction in cell viability [57,58].

## 3. Silver Nanoparticles

**Figure 4:**
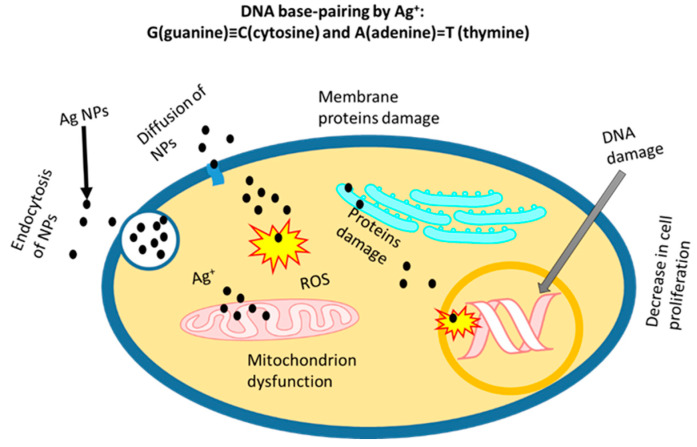
Cellular internalisation and cytotoxicity of Ag^+^ ion in the microenvironment.

Due to well-established antimicrobial properties, Ag NPs are widely used in many consumer products, such as toothpaste, shampoos, washing powders, kitchen utensils, toys, filters and deodorants [59,60]. It is very important to evaluate the potential toxicity for safe and effective use as applications of Ag NPs are rapidly expanding. In vitro studies of normal and tumour cell culture in the presence of Ag NPs can be performed for the comparison of potential toxicity and therapy. The cytotoxicity of prepared NPs varies due to the different synthesis methods and functionalisation of NPs. The best approach is to compare the cytotoxic effects on cellular components (in vitro studies) using naked Ag NPs. The effect of Ag NPs on different normal and tumour cell lines are summarised in Table 2. It is highly desired that NPs only induce toxicity in tumour cells, such as human liver cancer cells (HepG2), human breast cancer cell lines (MDA-MB 231), human embryonic kidneys (HEK293T) cells, human neuroblastomas (SH-SY5Y) cell line and others, whereas therapeutic or neutral effects are required in normal cells (e.g., human macrophages and human keratinocyte (HaCaT) cell lines). Ag^+^ addition results in modifications of DNA base pairs, deoxyribose fragmentations and DNA strand breakups. Due to the formation of two coordination complexes in DNA (high-energy and ground-energy states) by Ag^+^, a modification in the base pairs of DNA by a reaction with double and triple hydrogen bonds can be the result [61]. DNA base pairing and the changes in other organelles by Ag^+^ is shown in Figure 4. The Ag NPs can easily ionise to generate ROS to stimulate inflammatory responses through phagocytosis. Park et al. reported the generation of ROS in macrophage cells, and the activated macrophages increased the TNF-α secretion that led to cell membrane damage and apoptosis [62]. The green synthesis and surface coating of Ag NPs can reduce the toxic effect on healthy cells. Chitosan-coated Ag NPs exhibited good biocompatibility and efficient cellular internalisation in human embryonic cells (HEKs), as reported by Boca et al. [63]. The biogenic synthesis using different microorganisms where Ag^+^ is reduced to Ag^0^ in the presence of protein enzymes have been reported in several studies [64,65,66]. The Ag NPs synthesised by these methods showed increased biocompatibility, cellular uptake, antimicrobial properties and secretion [67]. The mechanisms of cellular internalisation and toxicity of Ag NPs are depicted in Figure 4.

**Figure 5:**
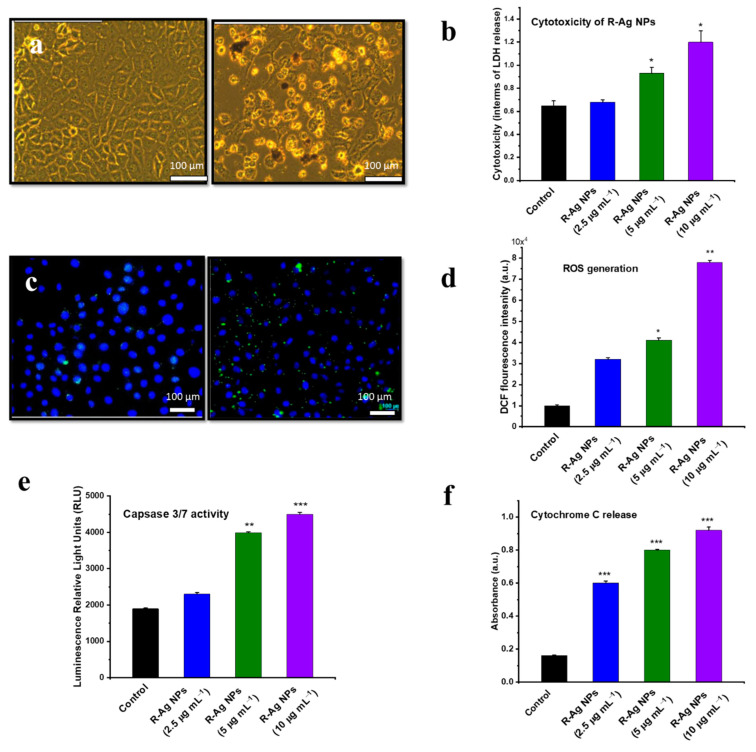
(**a**) Light microscopy images of MCF-7 cells depicting morphological changes after treatment with R-Ag NPs (10 µg mL^−1^) compared to control; (**b**) cytotoxicity of R-Ag NPs at 2.5, 5 and 10 µg mL^−1^ concentrations towards MCF-7 cells with reference to control measured by lactate dehydrogenase (LDH) activity assay; (**c**) fluorescent images of MCF-7 cells after treatment with R-Ag NPs at a 10 µg mL^−1^ concentration indicating the ROS presence compared to the control; (**d**) ROS generation in terms of DCF intensity measurements of treated cells at 2.5, 5 and 10 µg mL^−1^ concentrations of R-Ag NPs compared to the control; (**e**) caspase-3/7 activity measured as a function of caspase-dependent activity of treated cells for 24 h with reference to the control and (**f**) cytochrome-C activity measurement of treated cell with reference to the control. Where *** *p* ˂ 0.001, ** *p* ˂ 0.01 and * *p* ˂ 0.05. Images and graphs are reproduced with permission from [68], copyright 2018, Springer Nature, and represent the overall trend of results.

An in vitro study of Rubus-conjugated Ag NPs (RAg NPs) using an MCF-7 cell line exhibited increased cell death at higher concentrations (10 µg mL^−1^) compared to lower concentrations (5 µg mL^−1^) and the control (Figure 5a, reported by George et al.) [68]. The treatment with Ag NPs disrupted the mitochondrial respiratory chain reactions, which enhanced ROS generation, DNA damage and changes in ATP levels. An increased cytotoxicity determined by a lactate dehydrogenase (LDH) assay was observed in the treated cells (Figure 5b). Increased ROS generation was also witnessed in the dose-dependent NP treatment, as the highest number of ROS formations resulted during the 10 µg mL^−1^ treatment than at lower concentrations (Figure 5c,d). In the treated cells (MCF-7), cytochrome c and caspase-3/7 activity upregulation was observed compared to the control cells (Figure 5e,f) [68]. The present study concluded that RAg NPs enhanced ROS-dependent toxicity and cellular apoptosis in cancer cells [68].

**Table 2:** In vitro cytotoxicity with reference to size, shape and synthesis method of Ag NPs.

Cell Line	Shape and Size	Synthesis Method	Effect
Human mesenchymal stem cells	Spherical (<50 nm)	Commercial (Sigma-Aldrich, Steinheim, Germany)	Alteration at the chromosomal level, cytotoxicity and genotoxicity, oxidative stress and DNA damage [69,70].
Human hepatoblastoma (HepG2) cell line	Spherical (28–60 nm)	Citrate and polyvinyl alcohol-coated NPs	Increase in ROS production, depletion of GSH, oxidative stress and cytotoxicity [59,71].
Human macrophages			Generation of ROS and increase in oxidative stress, induction of heme oxygenase I, DNA damage [72].
Human neuroblastoma (SH-SY5Y) cell line	Spherical (30 nm)	Commercial (Shanghai Huzheng nano technology Co., Ltd., Shanghai, China)	Increased levels of phosphatase and tensin homolog, higher mitochondrial Ca^2+^ uptake, Ca^2+^ disrupts homeostasis and triggers apoptotic cell death [73].
Human umbilical vein endothelial cells (HUVECs)	Spherical (65 nm)	Reduction by citrate (Turkevich method)	Damages the cell membrane, oxidative stress and apoptosis resulted in dysfunction of cell [74].
Human embryonic kidney (HEK293T) cells	Spherical (60 nm)	Commercial (Sigma-Aldrich, Shanghai, China)	Cellular structural changes, downregulation of anti-apoptosis Bcl2-t and Bclw genes, upregulation of pro-apoptosis Bid gene, decreased cell viability and increased DNA damage [75].
Human breast cancer cell line (MDA-MB 231)	Spherical (20 nm)	Green synthesis (bacterium, B. funiculus)	Increased ROS generation and caspase-3 activation cause cellular apoptosis, oxidative stress and cytotoxicity [76].
Mouse MC3T3-E1 cell line	Spherical (100 nm, 50 nm)	Commercial (InkTec Co. (Asan, Korea), chemical reduction	Increased ROS generation, LDH release and expression of stress-related genes (ho-1 and MMP-3) and cellular apoptosis [77].
Rat PC12 cell line	Spherical (100 nm, 50 nm)	Commercial (InkTec Co. (Asan, Korea), chemical reduction	Intracellular ROS generation, lactate dehydrogenase release, changes in cell morphology and induced necrotic cell death [77].
Rat alveolar macrophage cell line	Spherical (15, 30, 55 nm)	Commercial (NovaCentrix Co., Austin, Texas, USA)	Mitochondrion depolarisation induced cytotoxicity, apoptosis and DNA damage [78].
Human Chang liver cells	Spherical (100, 28–30 nm)	Commercial (Sigma-Aldrich, St. Louis, MO, USA)	Protein carbonylation, lipid membrane peroxidation and DNA break [79].
Human keratinocyte (HaCaT) cell line	Spherical (25–50 nm)	Citrate reduction method and NP functionalisation	Long-lasting antiproliferative effect, even with a brief time contact with NPs. Permeation can damage the skin [80].

## 4. Manganese Oxide Nanostructures

Many metal and metal oxide NPs release toxic ions after cellular internalisation, depending upon the pH and the medium. The breakup of H_2_O_2_ or other species by NPs generate ROS, which lead to cellular oxidative stress [81]. An increase in oxidative stress in tumour cells causes cellular apoptosis and a decrease in proliferation. The oxidative burst is commonly employed to kill tumour cells. In the case of normal cells, oxidative stress is very dangerous because it can damage healthy cells. The toxic metal ions cause membrane leakage, inflammation, cell signalling cascades, protein damage and protein unfolding, DNA damage and mitochondrion dysfunction [82,83].

Manganese oxide (MnO_x_) exists in more than one oxidation state (+2, +3, +4, +6, and +7). Due to a variety of oxidation states, the compounds in oxidation state +3 and higher oxidation states are usually involved in redox reactions. Divalent and trivalent oxidation states are the most stable, whereas other oxidation states undergo redox changes. MnO_2_ finds applications as catalysts, magnetic resonance imaging, tissue imaging, biosensors, molecular adsorption, drug delivery and cancer immunotherapy [84,85]. The toxicity of manganese metal and its oxide varies and mainly depends upon the synthesis process, size, shape and oxidation state.

Manganese in an elemental form participates as a cofactor in certain enzymes, such as superoxide dismutase lyases, and tranferases [86]. In enzymatic activity, Mn^+3^ changes to Mn^+2^ during the dismutation of superoxide radicals into molecular oxygen, and catalysed by the SOD2 enzyme. Similarly, the superoxide and hydroxyl radicals dismutate into H_2_O_2_, and Mn^+3^ transforms into Mn^+2^ [87,88].

$${\mathrm{Mn}}^{+3}+{\;O}_{2}^{•-}\;\to {\;\mathrm{Mn}}^{+2}+O_{2}\;{\mathrm{Mn}}^{+2}+{\;O}_{2}^{•-}+H^{+}\;\to {\;\mathrm{Mn}}^{+3}+{\;H}_{2}O_{2}$$

However, an increased concentration might produce a neural disorder and adverse effect on human health. It has been reported that Mn-based NPs can enter dopaminergic neuronal cells and cause neurotoxicity [89]. Studies performed on breast cancer cells, human lung adenocarcinomas and glioblastomas reported the formation of ROS and LDH leakage [90]. Similarly, in a study on rat type-II epithelial cells, Mn_2_O_3_ NPs interacted with glutathione (GSH) and induced apoptosis [91]. A summary of Mn-based NPs’ effects on different cell lines is presented in Table 3. It can be observed from the data (Table 3) that Mn-based NPs produce neural toxicity, biochemical alterations, ROS, oxidative stress, cellular apoptosis and damage in cell membranes and other organelles. MnO crystals can also be used as contrast agents in T1- and T2-weighted images. T1-weighted image contrast relies on signal enhancement (positive effect), whereas T2-contrasting involves the reduction in signals (negative effect). T1 is more suited to the study of the morphological structure, and T2-contrasting is suited to pathological conditions. Mn-based NPs are theranostic agents; however, their toxicity must be carefully evaluated to suit their use for particular applications [92].

Recently, Mn-based composite nanostructures with improved biocompatibility, therapeutic and imaging capabilities have been synthesised. A dual-modality contrast agent MnWO_4_ nanostructure has been synthesised by Dong et al. [93]. The reported structure exhibited excellent dispersibility, biocompatibility and superior contrast efficacy, with applications as CT and T1-weighted MRI agents simultaneously.

**Figure 6:**
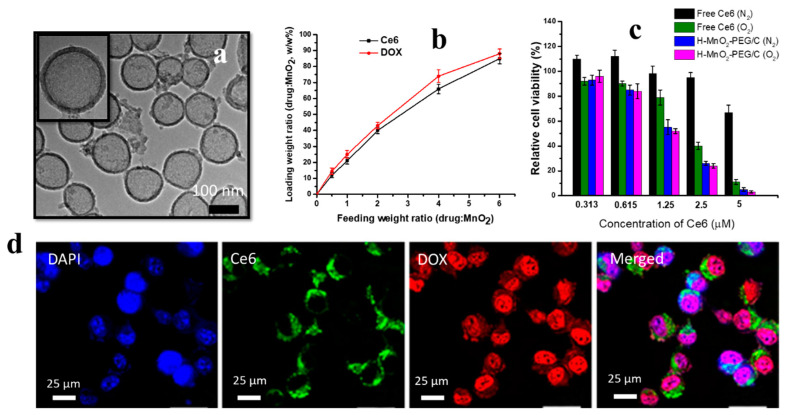
(**a**) A TEM image of H-MnO_2_ nanoshells, (**b**) loading-weight ratios of drugs (Ce6 and DOX) at different feeding weights, (**c**) in vitro PDT treatment of 4TI cells using Ce6 and/or H-MnO_2_-PEG/C irradiated under 660 nm light (5 mWcm^−2^ for 30 min) in N_2_ or O_2_ atmospheres and (**d**) confocal images of 4TI cells after 12 h of treatment with H-MnO_2_-PEG/Ce6&DOX, where blue, green and red indicate fluorescence in cells treated with DAPI, Ce6 and DOX, respectively. Data are represented as means ± sd (*n* = 5). Images and graphs are reproduced with permission from [94], copyright 2017, Springer Nature, and represent the overall trend of results.

There is an increased interest in the development of biodegradable nano-thernasotic platforms for antitumour applications. Yang et al. reported the synthesis of a hollow manganese dioxide (H-MnO_2_) nanosystem for controlled drug release and imaging [94]. A high-resolution TEM image (Figure 6a) shows polyethylene glycol (PEG) spherical H-MnO_2_ nanoparticles. The hollow NPs (H-MnO_2_-PEG) was further loaded with Ce6 and DOX drugs. The drug loading capability of H-MnO_2_-PEG NPs at different feeding rates of Ce6 and DOX is shown in Figure 6b. The treatment of 4TI cells with drug (Ce6)-loaded H-MnO_2_-PEG NPs in the presence of N_2_ and O_2_ after 660 nm light irradiation showed increased toxicity at increased drug concentrations (Figure 6c) [94]. It can be concluded from the toxicity data that H-MnO_2_-PEG NPs loaded with Ce6 can serve as effective PDT agents, even in a hypoxic environment. The confocal microscopy (Figure 6d) of 4TI cells treated with H-MnO_2_-PEG NPs loaded with Ce6 and DOX examined in blue, green and red channels (corresponding to DAPI, Ce6 and DOX, respectively) indicated the imaging capability of the developed system [94]. The developed biodegradable nanoplatform (H-MnO_2_-PEG NPs loaded with Ce6 and DOX) can be dissociated under reduced pH conditions and the tumour microenvironment (tumour hypoxia) to release loaded therapeutics, and can aid in the decomposition of cellular H_2_O_2_ to overcome tumour hypoxia to generate ROS [94].

**Table 3:** In vitro cellular behaviour with reference to size, shape and synthesis method of Mn-based nanostructures.

Cell Line	NP Size	Synthesis Method	Cell
Human breast cancer epithelial (MCF-7) cell line	MnO_2_, nano-flake (10–20 nm)	Hydrothermal processing	Oxidative stress mediated toxicity via p53 pathway, apoptosis induced by oxidative stress and dependent on dose [95].
Rat dopaminergic (N27) cells	Mn, unknown shape (25 nm)	Commercial (Quantum Sphere Pty Ltd., Dural NSW, Australia)	ROS generation, caspase-mediated proteolytic cleavage of proapoptotic protein kinase Cδ (PKCδ), loss of TH-positive dopaminergic neurons and induced autophagy [89].
Human fibrosarcoma epithelial (HT1080) cell line	MnO_2_, nano-flake (10–20 nm)	Hydrothermal processing	Higher NP internalisations, apoptosis induced by oxidative stress. Upregulation of pro-apoptotic genes and downregulation of anti-apoptotic genes [95].
PC-12 cells	Mn, cubic (40 nm)	Commercial (Nanotechnology, Inc, Austin, TX, USA)	Increased concentration of ROS, depletion of dopamine (DA) and its metabolites caused cellular toxicity [96].
Human lung epithelial (A549) cells	Mn_3_O_4_, unknown shape (10–20 nm)	Flame pyrolysis method	High NP internalisations, ROS generation induced oxidative stress and toxicity [14].
Human intestinal epithelial (Caco2) cells	Mn_3_O_4_, unknown shape (10–20 nm)	Flame pyrolysis method	Increased ROS generation caused oxidative stress and hence apoptosis and cell membrane damage [14,97].
Human neuroblastoma (SH-SY5Y) cell line	MnO_2_, round shape (40 nm)	Commercial (Research Nanomaterials, Inc., Houston, Texas, USA)	ROS generation induced oxidative stress, apoptosis (caspase-3 activation), PS translocation and fragmentation of chromosomes [98].
PC-12/rat pheochromocytoma cells	Elemental Mn, irregular shape (20 nm)	Unknown	Significant dopaminergic, neurotoxicity, upregulation of genes involved in DA metabolism and PA pathogenesis [87,99].
Rat lung epithelium (CCL-149) cell line	Mn_3_O_4_, sphere shape (30 nm)	Flame spray method	Redox disturbance, ROS generation, LDL release and cellular apoptosis [91].
Mouse fibroblast (L929) cells	MnO, irregular shape (15–25 nm)	High temperature pyrolysis	Activation of p53, increase in the bax and a decrease in bcl-2, leading to G2/M phase arrest, increase in caspase-3 activity and apoptosis [100].
Human cervical carcinoma (HeLa) cells	MnO, irregular shape (15–25 nm)	High temperature pyrolysis	ROS generation, loss of cell/cell contact between neighbouring cells, cytoplasm retraction, shrinkage of nuclei and multinucleated giant cells; cell death was observed [100].

## 5. Iron Oxide Nanostructures

Iron oxide is usually formed in two major forms (Fe II and Fe III) based on oxide structures, which include magnetite (Fe_3_O_4_), hematite (Fe_2_O_3_/α-Fe_2_O_3_) and maghemite (γ-Fe_2_O_3_). These oxides exist naturally in large quantities and are widely synthesised for their numerous applications. Fe_3_O_4_ and γ-Fe_2_O_3_ are extensively used in the field of biomedicine due to their paramagnetic/superparamagnetic nature and involvement in various biological processes. Iron oxide magnetic nanoparticles (IOMNPs) provide a theranostic platform where they can be exploited for diagnostic purposes, such as magnetic resonance imaging (MRI), and therapeutic purpose, such as drug delivery, magnetic cell separation, protein purification and bio-catalysts [101,102,103]. IOMNPs can be synthesised in different sizes and shapes using a variety of synthesis methods. Due to their widespread application and varying morphologies and surface properties, the toxicity of IOMNPs must be evaluated in relation to different conditions and body components [104]. The in vitro toxicity of IOMNPs varies with pH conditions and cell types. The Fenton reaction is a major ROS generating event leading to oxidative stress and cellular apoptosis. The Fenton reaction involves the reaction of Fe(II) with H_2_O_2_ to generate OH^−^ and OH^•^ radicals. The over production of ROS can cause damage to DNA and other cellular organelles (Table 4).

In several studies, it has been reported that tumour cells have high levels of ROS [105,106,107]. The major factors contributing to increased ROS might include oncogenic stimulation, mitochondrial malfunctions, increased metabolic activity and other dysregulated activities in the cells [105]. It appears advantageous that a high level of ROS favours the growth of cancer cells by activating several stress kinase pathways [108]. In addition, cancer cells can adopt high levels of ROS due to the presence of oncogene c-Myc, which increases the tolerance level by activating transcriptional genes for GSH biosynthesis in response to H_2_O_2_ [109]. It is apparent that increased levels of ROS and oncogenic transformation result in the increased sensitivity of the cells to generate ROS [110]. Several chemicals and nanomaterials, such as phenylethyl isothiocyanate, titanium-based materials and piperlongumine increase ROS levels and selectively target tumour cells, and do not cause much damage to normal cells [106,110]. On the other hand, for increased ROS levels, cells are treated with certain chemicals, such as N-acetyl-l-cysteine, to scavenge ROS [111]. Thus, selective treatment with ROS generation/scavenging in malignant cells while protecting normal cells is an effective method for cancer therapy.

**Figure 7:**
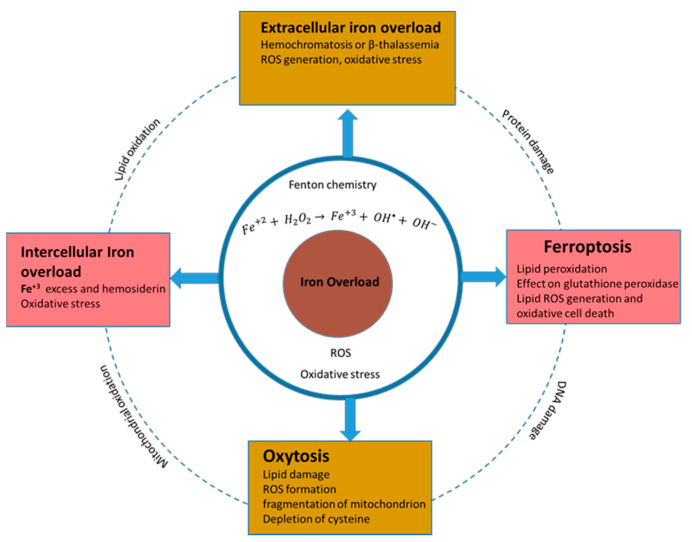
Common Fe cytotoxicity mechanisms in the microenvironment.

Iron is an important factor in the ROS-linked homeostasis of normal cells and can cause dysregulation to result in tumorigenesis. Iron regulates different functions in cells of different tissues in the body. The common Fe-related phenomena and their in vitro effects are presented in Figure 7, and are also described as follows:

- **Ferroptosis**

Iron-dependent oxidative cell death is termed as ferroptosis. It is triggered by structurally different small molecules, such as erastin, RSL3 and sulfasalazine. Ferroptosis is different from apoptosis, autophagy and other forms of necrosis [112,113]. The fundamental details of iron oxide involvement in ferroptosis are not clear. It is hypothesised that the inhibition of cysteine uptake causes the depletion of the endogenous antioxidant tripeptide glutathione, which causes the accumulation of iron-dependent ROS and leads to cell death [112,114]. Ferroptosis can be prevented by using iron chelators (such as deferoxamine and lipophilic antioxidants, i.e., vitamin E and Trolox) [112,115].

- **Oxytosis**

Oxytosis or oxidative glutamate toxicity is linked to iron and is observed in certain brain cells in the absence of cystine [116]. Oxytosis in neural cells initially involves membrane lipid damage and results in cell death, including LOX activation, Ca^2+^ influx into the cell, overproduction of mitochondrial ROS and fragmentation of mitochondrion [113,114,116]. Iron-dependent oxidative cell death was observed in premature oligodendrocytes cells using high concentrations of glutamate or having the absence of cysteine. In the nervous system, the depletion of premyelinating cells causes periventricular leukomalacia (PVL) disease, which is characterised by white-matter lesions within the brain and acts as a precursor for cerebral palsy development [117,118].

- **Intercellular iron accumulation as a mediator of cell death**

According to the research reports in recent years, liver toxicity is caused by iron overload and can be ameliorated by mitochondrially targeted oxidation [119]. It is important to understand that high levels of iron are not always harmful for cell viability and proliferation because, sometimes, iron overload contribute to cell death in certain cell types and tissues, and it may increase cell proliferation and viability. Antiproliferative and proliferative functions can also be performed through cell-nonautonomous or cell-autonomous effects on cell mutation and tissue microenvironments, cell signalling and iron-dependent enzyme function. For example, Parkinson’s disease is linked to the depletion of the Tau protein (linked to iron export) by iron accumulation [114,120].

Neurodegenerative disease are increasing rapidly; they are associated with iron accumulation within neurons [121]. An accumulation of iron generates ROS through enhanced Fenton chemistry mediation. In Parkinson’s disease, dopaminergic neuronal populations are susceptible to degeneration and involve increased amounts of mitochondrion ROS [122,123]. Iron chelators can be employed to prevent high levels of ROS production via the autophagy of ROS producing mitochondrion. Thus, iron chelators or ROS scavengers lower the damaging levels of ROS and, hence, oxidative stress [122].

Iron dyshomeostasis is the common factor in different neurodegenerative diseases, such as AD, frontotemporal dementia (FTD) and Lewy body dementia. Iron promotes the aggregation and pathogenicity of the β-amyloid peptide, α-synuclein, TDP43 and tau protein [124,125,126].

- **Extracellular iron accumulation as a mediator of cell death**

Iron acts as mediator for signal responses in the excitotoxic death of cortical neuronal populations of a mouse in response to N-methyl-d-aspartate (NMDA). The required iron is transported from the outside of the cell by an iron transporter divalent metal transporter 1 (DMT1) [127]. An analysis of NDMA treatment results show that increased ROS production and oxidative stress induce cell death. It is quite obvious that iron import and NOX-derived ROS production enhance ROS-mediated neural cell deaths [114,128]. The common phenomena of cellular toxicity due to iron overload and Fenton chemistry are depicted in Figure 7.

**Table 4:** In vitro cellular effect with reference to size, shape and synthesis method of Fe_2_O_3_ and Fe_3_O_4_ nanostructures.

Cell Line	NP Size	Synthesis Method	Effect
Human hepatocyte (HL-7702) cell lines	Fe_3_O_4_, unknown shape (50 nm)	Commercial (Colorobbia Consulting-Cericol, Vinci, Italy)	Induction of apoptosis and autophagy, nuclear condensation and chromosomal DNA fragmentation were observed [129].
Human hepatoma HepG2 cells	Fe_2_O_3_, spherical (50 nm)	Commercial	Mitochondrial apoptosis through activation of loop phosphorylation, release of cytochrome c from the mitochondria, decrease in Bcl-2 protein expression, PARP activation and caspase cascades, ROS generation and DNA damage [130].
Human lung (BEAS-2B) cells	Fe_3_O_4,_ Fe_2_O_3_ irregular shape (˂100 nm)	Commercial (Sigma-Aldrich, St. Louis, USA)	Increased ROS generation and oxidative stress, mitochondrion and DNA damage [131].
Human cerebral endothelial cells (HCECs)	Fe_3_O_4_, unknown shape (9 nm)	Commercial (PlasmaChem GmbH, Berlin, Germany)	Overexpression of cathepsin D accelerated apoptosis, ROS generation transported into lysosomes interfering with the lysosomal hydrolases, cathepsins D and B, and induced oxidative stress and, hence, autophagy [132].
Human lung cancer (A549) cell line	Fe_3_O_4,_ Fe_2_O_3_ irregular shape (˂100 nm)	Commercial (Sigma-Aldrich, St. Louis, MO, USA)	ROS generation, increased oxidative stress, cellular apoptosis and DNA damage [131].
Lung cancer (HCC827) cell line	Iron oxide, unknown shape (NPs (73 nm)	Co-precipitation method and NP conjugation	Reduced EGFR phosphorylation, increased γH2AX foci and induced apoptosis, which resulted in suppression of tumour growth [133].
Chinese hamster ovary (H9T3) cell lines	Fe_2_O_3_, hexagonal shape, (20–30 nm)	Harvard Versatile Engineered Nanomaterial Generation System (VENGES)	Cellular apoptosis and double-stranded DNA breaks [134].
Human fibrosarcoma (HT-1080) cells	Irregular and spherical shapes, Fe_3_O_4_ (10–150 nm)	Massart’s method, and NP coating	Increased ROS generation caused oxidative stress and lipid peroxidation. Oxidative damage induced DNA damage [135].

## 6. Recently Developed Theranostic Nanostructures

Over the past decade, various inorganic nanostructures and quantum structures have been developed and investigated for thernostics applications. In particular, superparamagnetic NPs, silicon spheres, transition metals and lanthanide-based quantum structures are of interest for biological applications. The development of advanced size- and shape-controlled nanotheranostics require novel or modified synthesis techniques. As we know, synthesis techniques play an important role in the surface property tuning, size and shape of NPs. Nanodots or quantum dots (sized 2–20 nm) offer a wide range of properties in diverse fields of applications due to their extremely high surface area, stability and efficient catalytic, magnetic and electrical properties. Transition metals and lanthanide series elements are the focus of current research for the development of novel nano/quantum structures. Synthesis conditions can stabilise the new phases in the formed quantum structures. It can also be very convenient to introduce oxygen deficiency to the developed nanostructures. 

## 7. Maghemite Nano- and Quantum Structures

**Figure 8:**
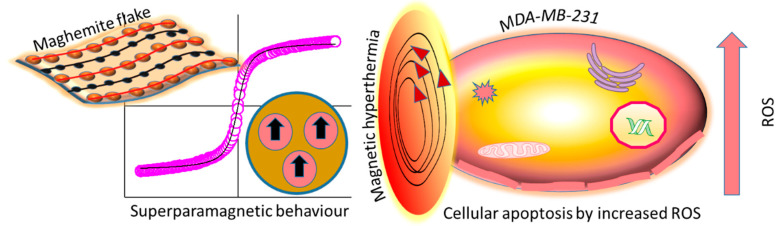
In vitro cellular cytotoxicity of theranostic superparamagnetic γ-Fe_2_O_3_ quantum flakes in MDA-MB-231 cell line through augmentation of ROS, whilst also having excellent magnetic hyperthermia and fluorescence imaging capabilities. Image reproduced with permission from [141], copyright 2021, Royal Society of Chemistry.

The properties of superparamagnetic iron oxide nanoparticles (SPIONs) depend upon size, shape and iron oxide structure. The particle/crystal size and shape affect the NPs’ internalisation, retention, circulation and toxicity [136,137]. It has been shown that rod-shaped NPs present higher toxicity than sphere-shaped NPs. [138,139]. The oxidation state (Fe^+3^, Fe^+2^) in SPIONs also affects in vitro and in vivo cytotoxicities. In many studies, it has been shown that γ-Fe_2_O_3_-nano/quantum structures did not cause much toxicity or DNA damage (Table 5), and exhibited superparamagnetic behaviour, Figure 8 [138,140].

Recently, quantum dots have gained much interest due to their broad range of excitation and absorption properties, high surface area, excellent photoluminescence and photostability. When the size of magnetic NPs reaches < 10 nm, they start behaving as a single magnetic domain with superparamagnetic behaviour [142,143,144,145]. Quantum structures can be employed as fluorescent agents in in vitro and in vivo biomedical imaging [146].

Among iron oxide-based nanostructures, γ-Fe_2_O_3_ has excellent magnetic properties, biocompatibility and pH-dependent selective toxicity [141,147]. It has been reported that γ-Fe_2_O_3_ can be employed as an effective nanozyme for both peroxidases, such as activity (at acidic pH), and catalases, such as mimetic activity (at neutral pH) [141,147]. 

**Figure 9:**
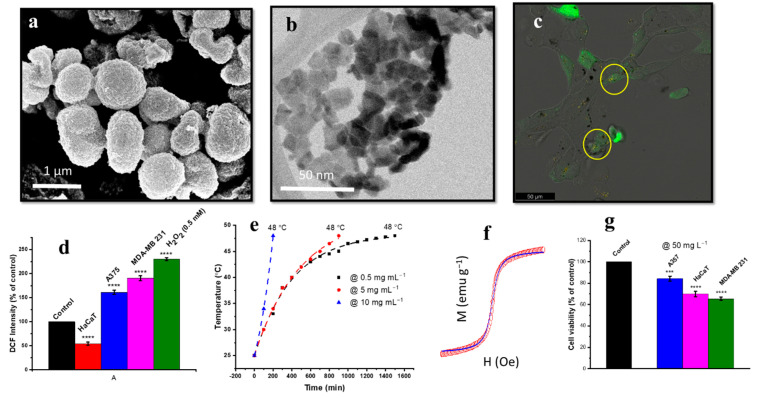
(**a**) SEM image of γ-Fe_2_O_3_ quantum structure; (**b**) TEM image of γ-Fe_2_O_3_ quantum flake (MQF); (**c**) confocal overlay image of MDA-MB-231 cell line with NPs internalisation; (**d**) measurement of ROS scavenging in HaCaT, A375 and MDA-MB-231 cell line with reference to the positive and negative controls; (**e**) hyperthermic ability of MQFs in water using 0.5 mg L^−1^, 5 mg L^−1^ and 10 mg L^−1^ concentrations of MQFs; (**f**) superparamagnetic behaviour of MQFs at room temperature; (**g**) cell viability (HaCaT, A375 and MDA-MB-231 cell line) at 50 mg L^−1^ MQF concentration with respect to the control. Where **** *p* < 0.0001, *** *p* < 0.001, ** *p* ˂ 0.01 and * *p* ˂ 0.05. Pictures are reproduced with permission from [141], copyright 2021, Royal Society of Chemistry, and represent an overview and general trend of results.

Rehman et al. reported the synthesis of micron-sized γ-Fe_2_O_3_ flowers (Figure 9a) using hydrothermal processing for biological applications, where individual flowers consisted of quantum-sized 2D flakes (Figure 9b) [141]. These quantum flakes exhibited superparamagnetic properties (Figure 9f) and showed excellent hyperthermia properties (Figure 9e) for tumour cells (MDA-MB-231). Cell viability data showed higher toxicity in malignant cells (MDA-MB-231 and A375) than non-malignant cells (HaCaT) (Figure 9g). The ROS scavenging/generation properties of γ-Fe_2_O_3_ quantum flakes (MQFs) compared to the negative (cells only) and positive (H_2_O_2_) controls are presented in Figure 9d. An increased level of ROS was observed in MDA-MB-231 and A375 cells compared to HaCaT cells. A higher cellular uptake of MQFs was also observed in malignant cells compared to non-malignant cells, as reported in the present study [141].

These magnetic quantum dots (MQDs) are capable of inhibiting tumour growth by ROS augmentation to induce selective toxicity (Figure 8, Table 5) [141,143,148,149]. 

**Table 5:** Size, shape and synthesis method of γ-Fe_2_O_3_ NPs and their in vitro biological effect on malignant and non-malignant cell lines.

Cell Line	NP Size and Shape	Synthesis Method	Effect
He La cells	γ-Fe_2_O_3_, spherical shape (<20 nm)	Microwave-assisted hydrothermal	No significant toxicity, generation of ROS, enhanced oxidative stress, application as multimodal (ROS and hyperthermia) anticancer therapy [138].
Human breast cancer line (MDA-MB 231)	γ-Fe_2_O_3_, 2D flakes (4–10 nm)	Hydrothermal processing and calcination	ROS generation, apoptosis by magnetic hyperthermia therapy, higher cellular internalisation of flakes [141].
Human keratinocyte cell line (HaCaT)	γ-Fe_2_O_3_, flakes (4–10 nm)	Hydrothermal processing and calcination	Less γ- Fe_2_O_3_ flake uptake by HaCaT cells than malignant (MDA-MB 231) cells, less apoptosis by magnetic hyperthermia therapy [141].
Human melanoma cell line (A375),	γ-Fe_2_O_3_, 2D flakes (4–10 nm)	Hydrothermal processing and calcination	ROS generation, higher γ-Fe_2_O_3_ flake uptake by A375 cells. Apoptosis due to ROS augmentation and magnetic hyperthermia therapy [141].
Human prostate cancer (PC3) cell line	γ-Fe_2_O_3_, nanosphere (12.5 nm)	Co-precipitation method	Lower NP uptake, lower toxicity than Fe_3_O_4_ NPs [150].

## 8. Indium Tin Oxide (ITO) Nanostructure in the Biological Field

**Figure 10:**
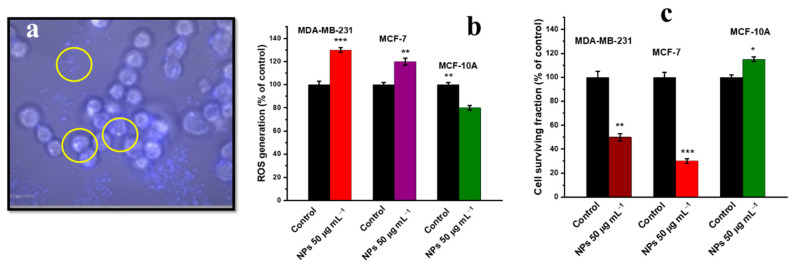
(**a**) Fluorescent microscopy images of MDA-MB-231 cell line after treatment with ITO NPs for 24 h; (**b**) ROS generation/scavenging in MDA-MB-231, MCF-7 and MCF-10A cell lines; (**c**) cell viability of MDA-MB-231, MCF-7 and MCF-10A cell lines after treatment with IT0 NPs at 50 μg mL^−1^ concentration, where *** *p* ˂ 0.001, ** *p* ˂ 0.01 and * *p* ˂ 0.05 with respect to the control. Pictures are reproduced with permission from [157], copyright 2021, American Chemical Society, and show only an overview of obtained results.

In the last few years, ITO remained one of the most researched materials for a variety of applications. It is an n-type semiconductor with a band gap of 3.2 eV [151,152]. It finds its main applications as liquid crystal display devices, electrochromic cells, solar cells and sensor modules [151,153]. There is limited research on ITO NPs for biomedical applications. In the biomedical field, it is mostly employed in biosensors, where they are exploited in the electrochemistry of biomolecules and for the immobilisation of immunoreagents [154]. Hu et al. reported a glucose biosensor based on an ITO/Au NP bilayer composite, which showed excellent electrochemical behaviour [155]. This sensor exhibited high sensitivity to the detection of the HIV virus in a label-free system with only a small amount of reagent required, and also showed high stability. In many studies, ITO was reported as a substrate layer with other metal oxide NPs (such as ZnO, Au, Ag, Pt, Ni, Fe_2_O_3_ NPs) for the development of biosensors for a range of biomedical applications [156]. Recently, ITO NPs have been reported by Hsu et al. as theranostic agents for the treatment of cancer, Figure 10 [157]. Oxygen-deficient ITO NPs were prepared by the chemical precipitation method, followed by calcination at 400 °C [157]. The modification of the surface structure affects the physiochemical properties of NPs. ITO NPs can be employed in biomedical imaging due to their intrinsic fluorescent properties. The band gap and quantum size effect can also play important roles in the fluorescent-related imaging of ITO [157,158,159].

**Figure 11:**
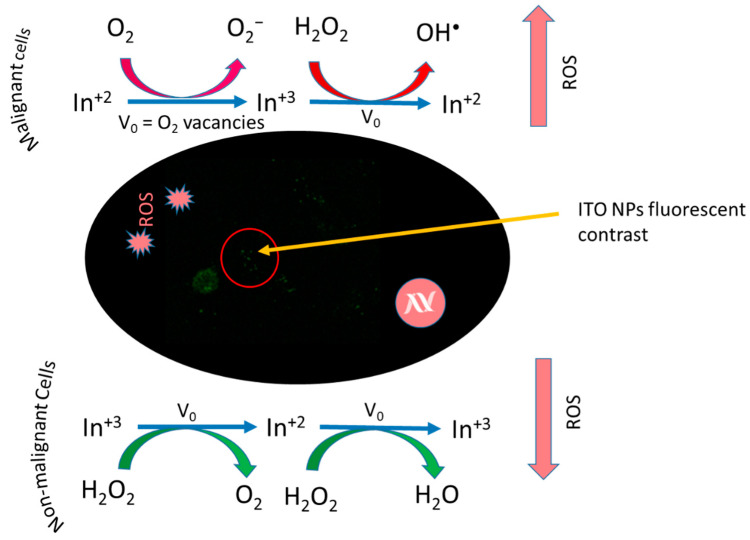
ITO nanotheranostic structure with intrinsic fluorescent and ROS scavenging properties.

The reported in vitro cytotoxicity study of ITO NPs on human epithelial (MDA-MB-231) cells, breast cancer (MCF-7) cells and human epithelial (MCF-10A) normal breast cells is summarised in Table 6, and the mechanism is shown in Figure 11 [157]. According to the results, ITO NPs induced selective toxicity in MDA-MB-231 and MCF-7 cell lines by ROS generation, which caused higher oxidative stress in comparison to non-malignant MCF-10A cell lines, shown in Figure 10b,c. Fluorescence microscopy revealed ITO NP internalisation, as shown in Figure 10a. The authors found that ITO NPs not only generated/scavenged ROS depending upon the tumour microenvironment, but also provided fluorescent imaging capabilities in in vitro assays. ITO-based NP systems can provide successful selective tumour treatment if synthesised through controlled or functionalised parameters [157].

The study also featured the fluorescent and anatomical contrast properties of the NPs (Figure 11) for computed tomography (CT) imaging. The study proposed that ITO NPs can be theranostic agents for the selective treatment of cancer based upon oxidative stress augmentation and imaging capability [157]. 

## 9. Doped Cerium and Nanodot-Encrusted Structures

**Figure 12:**
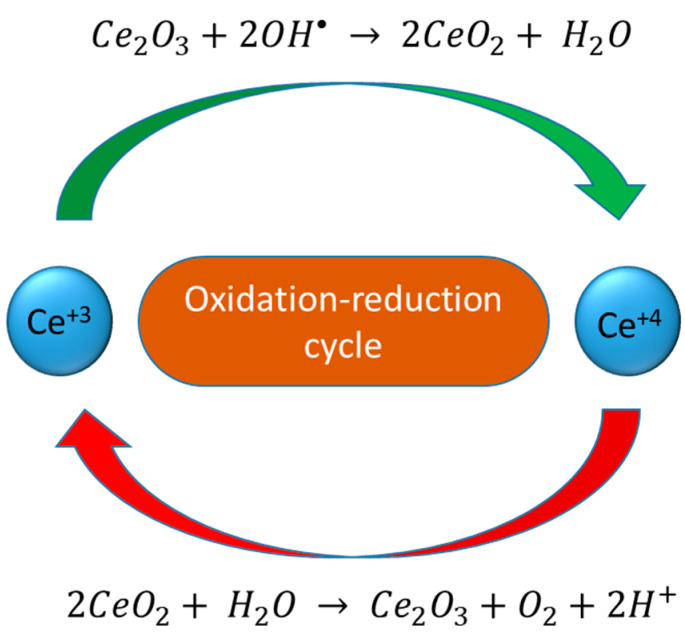
Redox cycle of cerium nanostructure containing Ce_2_O_3_ and CeO_2_ for ROS scavenging.

**Figure 13:**
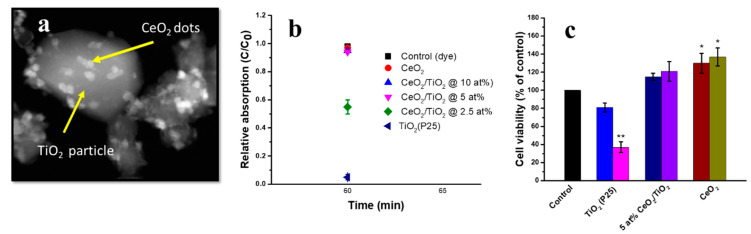
(**a**) Dark-field TEM image of CeO_2_ nanodot-encrusted TiO_2_, (**b**) degradation experiment of CeO_2_/TiO_2_ structure at different atomic percentages (at%) of CeO_2_ in comparison to P25 and dye (crystal violet)-only degradation and (**c**) HaCaT cell viability of CeO_2_/TiO_2_ @5 at% CeO_2_ in comparison to positive (cell only) and negative (P25) controls after 15 min of UV exposure. Where ** *p* ˂ 0.01, * *p* ˂ 0.05. Pictures are reproduced with permission from [175], copyright 2020, Royal Society of Chemistry, and represent the general trend of results.

Cerium (Ce) is a lanthanide series element with 4*f* shielded electrons, which contribute to excellent physiochemical properties. The most common oxidation states of Ce are Ce^+3^ and Ce^+4^. [160]. Due to the special electronic configuration and reversible switching between oxidation states (Ce^+3^ and Ce^+4^), it is an attractive material for many applications, such as UV absorption, fuel cells, fuel oxidation, catalysis, polishing and others [161,162,163,164]. In the biomedical field, cerium oxide NPs have attracted special interest due to their regenerative and multi-enzymatic properties for ROS scavenging [163,165]. Bulk cerium oxide mainly consists of Ce^+4^, whereas in nano form, a considerable amount of Ce^+3^ is present, which enhances its catalytic properties and biological role [166,167]. Cerium NPs have been studied in different cell lines and animals, and they exhibited biocompatibility. Cerium NPs have advantages over other NP systems due to their wide range of therapeutic and imaging capabilities. The major factor that contributes towards the wide range of biomedical properties in cerium nanostructures is the reversible switching between Ce^+4^ and Ce^+3^ oxidation states [168]. An increased level of Ce^+3^ in the CeO_2_ nanostructure enhances the photocatalytic activity by reducing the band gap of CeO_2_. Oxygen vacancies help to increase the level of Ce^+3^ by preventing the recombination of charge centres. Regenerative nano-ceria oxidation-reduction cycles for ROS scavenging proceed, as presented in Figure 12: 

Due to the enzymatic activity of cerium oxide NPs, it has recently been explored for the sensing of molecules in a biological environment. Cerium NPs can be employed as inorganic probes for the detection of H_2_O_2_, dopamine, glutamate polyphenols and glucose, and they can replace soluble organic redox dyes, oxidase and peroxidase enzymes [168,169,170]. In relation to fluorescence imaging, cerium NPs have weak fluorescence, which can be improved by doping with highly fluorescence elements, such as europium (Eu^+3^) [171,172]. Likewise, the magnetic properties of cerium NPs can also be improved by doping with certain magnetic elements, such as gadolinium (Gd), dysprosium (Dy) and holonium (Ho) for MRI applications [163,173,174]. Other interesting approaches, such as core shell and cerium nanodots or quantum dot-encrusted structures have been reported to create composite theranostic nanostructures. Morlando et al. reported cerium nanodot-encrusted titania (TiO_2_) for UV absorption and biocompatible ROS scavenging applications [175]. Ceria-encrusted TiO_2_ NPs were synthesised by a combination of thermal precipitation and chemical precipitation approaches [176]. The reported nanostructure showed biocompatibility, high UV absorption and ROS scavenging [176]. Morlando et al. synthesised cerium nanodot-encrusted rutile TiO_2_ rods by hydrothermal processing [177]. The encrustation of rutile TiO_2_ rods increased the biocompatibility and lowered the photocatalytic activity of TiO_2_, and resulted in high UV absorption [177]. In another study conducted by Morlando et al., CeO_2_ nanodot-encrusted TiO_2_ NPs were synthesised using the precipitation technique [175]. The morphology of the synthesised encrusted nanostructure studied by TEM is presented in Figure 13a. The presence of CeO_2_ nanodots on the surface facilitated ROS scavenging by reducing the photocatalytic activity of TiO_2_ NPs. The synthesised nanostructure exhibited a very negligible dye degradation at 5 and 10 atomic% of CeO_2_ compared to TiO_2_ NPs due to reduced photocatalytic activity (Figure 13b). High cell viability (HaCaT cell line) was also observed for the encrusted TiO_2_ NPs (5 and 10 atomic% of CeO_2_) with reference to the positive control (TiO_2_ NPs), which was slightly lower than CeO_2_ NPs (Figure 13c) [175].

**Figure 14:**
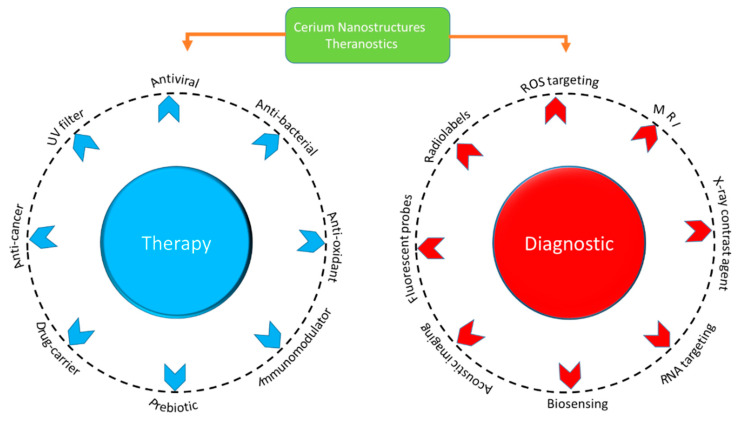
Therapeutic and diagnostic applications of cerium-based nanostructures.

Thus, cerium oxide NPs have emerged as powerful antioxidants and have been reported for their multi-enzymatic mimetic activity, and efficient ROS and NOS scavenging properties. The theranostic applications of Ce-based nanostructures are detailed in Figure 14.

## 10. Oxygen-Deficient Lanthanum Oxide

Lanthanum (La) is a rare-earth (RE) element and is finding many technological and industrial applications in the form of lanthanum oxide (La_2_O_3_), such as chemical catalysts, laser material, precision optical glasses, electrode material, light-emitting material (blue powder) and hydrogen storage material [178,179]. It is also employed in many biomedical applications, such as targeted drug delivery within the body, suppression of bacteria and viruses [180,181,182], binding agent for several proteins, calcium channel suppression, fluorescence dyes and ROS scavenging activity [181,183]. In the electronic configuration of La, 6*S* electrons are drawn inward due to the poor shielding of the nuclear charge by 4*f* electrons and results in the contraction of the atomic radius. The filling of the 4*f* shell increases with the increase in the atomic number, whereas 5*d*^1^ configuration appears in La, Ce, Gd and Lu [184,185,186]. The availability of 4*f* and 5*d* electrons for reaction imparts unique magnetic, fluorescent, electrical and catalytic properties in rare-earth oxides [182].

**Figure 15:**
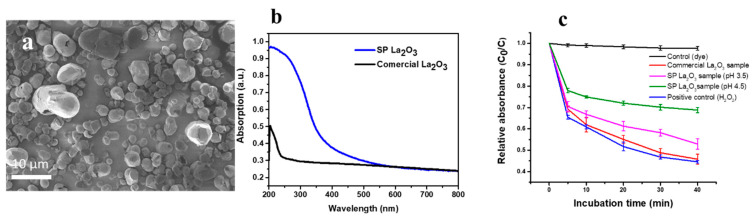
(**a**) SEM imaging of spray pyrolyzed La_2_O_3_, (**b**) UV absorption properties of spraypyrolyzed La_2_O_3_ in comparison to commercial La_2_O_3_ in the range of 200–450 nm and (**c**) degradation assays to assess the ROS generation by La_2_O_3_ NPs using crystal violet (CV) dye. Pictures are reproduced with permission from [187], copyright 2020, Springer Nature, and represent the general trend of results.

The removal of surface oxygen atoms from the surface of La_2_O_3_ can affect the oxidation state, catalytic properties and biocompatibility. Rehman et al. reported oxygen-deficient La_2_O_3_ synthesis through the spray pyrolysis method [187]. SEM imaging (Figure 15a) reveals the hollow, sphere-like morphology of the synthesised nanostructure, where each individual sphere consists of nanocrystals. The nanostructure recorded a higher UV-Vis absorption than commercial La_2_O_3_ (Figure 15b). One interesting feature of the nanostructure is its ROS scavenging property, as shown in Figure 15c, which indicates higher ROS scavenging activity at pH 4.5 than pH 3.5 compared to the negative (dye only) and positive (H_2_O_2_) controls. The reported structure exhibited excellent ROS scavenging activity in a concentrated H_2_O_2_ environment. The nanostructure exhibited a decreased bandgap, high UV absorption and antioxidant behaviour compared to commercial La_2_O_3_ [187]. The nanostructures based on La_2_O_3_ can be exploited for ROS scavenging applications if synthesised under controlled processing conditions.

Liu et al. reported the antioxidant activity of La^+3^ in the roots of rice seedlings. The internalisation of La^+3^ was observed by laser scanning confocal microscopy. A H_2_DCFDA probe was employed to study the change in ROS concentration [188]. The results of this study show a decrease in ROS level at a concentration of 0.05 mM of La^+3^ [157]. Wang et al. reported the ROS scavenging mechanism and protection effect against oxidative stress in soybean seeds [189].

**Figure 16:**
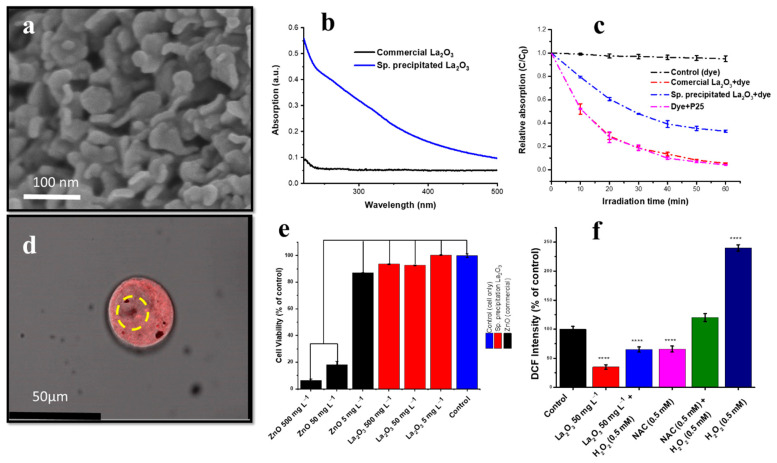
(**a**) SEM image of La_2_O_3_ NPs synthesised by spray precipitation (Sp. Precipitation) method, (**b**) UV absorption properties of spray-precipitated sample in comparison to commercial La_2_O_3_ in the range of 210–500 nm, (**c**) UV degradation of synthesised sample in comparison to P25 (photocatalyst) and commercial sample, (**d**) fluorescence microscopy of La_2_O_3_ NPs’ cellular internalisation in HaCaT cell line, (**e**) cell viability of HaCaT cell line in comparison to ZnO NPs and control and (**f**) ROS scavenging assay in terms of DCF fluorescence with reference to NAC and H_2_O_2_ assays. Where **** *p* ˂ 0.0001, *** *p* ˂ 0.001, ** *p* ˂ 0.01 and * *p* ˂ 0.05 with respect to the control. Pictures are reproduced with permission from [182], copyright 2021, American Chemical Society, and represent the general trend of results.

**Figure 17:**
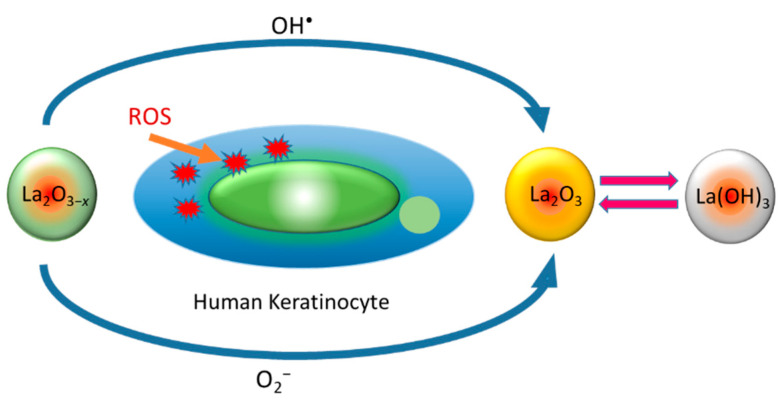
Interconversion of oxygen-deficient La_2_O_3−x_, La(OH)_3_ and La_2_O_3_ for ROS scavenging in HaCaT cells.

The La_2_O_3_ nanostructure synthesised by the spray precipitation method for biological applications was reported by Rehman et al. [182]. Mostly, an irregular or thick plate-like morphology was observed in the SEM imaging (Figure 16a). The UV absorption measurements of the spray-precipitated nanostructure (La_2_O_3_) showed higher absorption in the 200–500 nm range than the commercial sample. The dye degradation assay in the presence of a P25 photocatalyst exhibited a protective effect against ROS generation through scavenging. A lower dye degradation (~44%) was recorded in the presence of La_2_O_3_ NPs compared to the P25-only assay (~96%) shown in Figure 16c. A confocal microscopy study showed the cellular (HaCaT cell line) internalisation of NPs (Figure 16d) [182]. The cytotoxicity data revealed the biocompatible nature of the nanostructure, even at a higher concentration (500 mg L^−1^) with reference to ZnO (Figure 16e). Antioxidant property evaluations by the DCF fluorescent assay on HaCaT cells with reference to NAC and H_2_O_2_ (positive control) are shown in Figure 16f. A higher ROS scavenging was observed in La_2_O_3_ NP-treated cells than NAC-treated cells compared to the positive control. ROS scavenging in non-malignant HaCaT cells lowered ROS-induced oxidative stress [182]. The most dominating mechanism of ROS scavenging is the interconversion of La_2_O_3−x_, La(OH)_3_ and La_2_O_3_ is shown in Figure 17.

## 11. Tantalum Oxide Nanostructures

Tantalum oxide (Ta_2_O_5_)-based nanostructures are being employed in CT contrast in lab scale diagnoses [190]. Materials with high Z and density (*ρ)* are considered suitable to achieve a better resolution in CT imaging. A tantalum (Ta) nanostructure can absorb more X-rays due to high Z and *ρ* (75, 16.4 g cm^−3^) and can be an excellent choice for a high-performance contrast agent. The choice of Ta_2_O_5_ NPs for a contrast agent is based on its biocompatible nature, compared with Au NPs, because Au NPs also offer an excellent choice for radiosensitisation, but, at the same time, they are toxic to many cell lines and tissues (Table 7) [190,191]. Thus, Ta_2_O_5_ NPs exhibited biocompatible and theranostic properties in cell culture studies.

**Figure 18:**
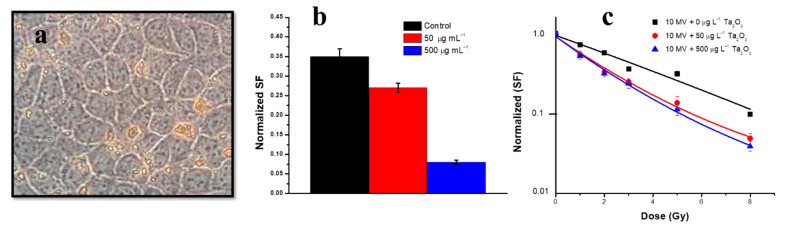
(**a**) Ta_2_O_5_ NP distributions in MDCK cells (studied by light microscopy), (**b**) gliosarcoma cell line survival factor after treatment with Ta_2_O_5_ NPs in comparison to the control and (**c**) 9L cell survival curves after irradiation with 10 MV x-rays and dose enhancement in the presence of 50 μg L^−1^ and 500 μg L^−1^ in comparison to cells only (control). Pictures are reproduced with permission from [192,193], copyright 2016, Elsevier, and represent an overview and general trend of results.

Engels et al. studied Ta_2_O_5_ NPs for radiation dose enhancement to treat the tumour cells. In the MDCK cell line, Ta_2_O_5_ NPs were internalised (black-brown spots) successfully, as shown in Figure 18a, observed by light microscopy [192]. Another tumour cell line (9L gliosarcoma) was irradiated with a radiation beam of energy 90 keV with a specific width and pitch and at 50 µg mL^−1^ and 500 µg mL^−1^ concentrations, compared to the control for the purpose of radiation dose enhancement (Figure 18b,c). It can be seen that “cell survival fraction” is at a minimum level at a high NP dose (500 µg m L^−1^) with reference to control cells. NPs treated with 500 µg mL^−1^ showed a lower cell survival fraction compared to the control than lower NP concentrations (50 µg mL^−1^) when irradiated with 10 MV X-ray beams [193].

Oxygen-deficient tantalum oxide (TaO*_x_*) offers a better choice than Ta_2_O_5_ for higher CT visualisation, as reported by Chakravarty et al. [194]. Depending upon the *x* value, TaO*_x_* has higher concentration of Ta and higher density than Ta_2_O_5_ [194]. Both the higher density and higher concentrations of Ta aid in greater X-ray attenuations to result in higher CT contrasts. The development of TaO*x* nanocrystals was first reported by Hyeon et al. synthesised through the sol–gel method using the precursor tantalum (v) ethoxide [195].

**Table 7:** In vitro cellular behaviour of Ta_2_O_3_ and TaO_x_ with reference to size, shape and synthesis method.

Cell Line	NP Size and Shape	Synthesis Method	Effect
HEK 293 cell line	TaO*_x_* nanocrystals (9–12 nm)	Sol–gel	No significant cytosolic dissolution under cytosolic and lysosomal conditions, high in vitro cell viability [194].
RAW 264.7 macrophage cells	TaO*_x_* nanocrystals (9–12) nm	Sol–gel	No significant cell toxicity, and produced effective CT contrast [194].
RAW 264.7 macrophage cells	TaO*_x_* NPs (5–15 nm)	Micro-emulsion	Maintained high in vitro cell viability, effective in vitro fluorescence and CT contrast [195].
Rodent brain cells (9L gliosarcoma cancer cells)	Ta_2_O_3_, irregular (50–70 nm)	Precipitation and calcination	Non-toxic over a wide range of concentrations. cell death in the localised radiation therapy and due to radiosensitivity of 9L cells [191].
Madin–Darby Canine Kidney (MDCK)	Ta_2_O_3_, irregular (50–70 nm)	Precipitation and calcination	Non-toxic over a wide range of concentrations [191].
Mammalian (HeLa) cell line	TaO*_x_* core shell structure (~10 nm)	Aqueous sol–gel method-based reverse-micelles assembly	Less toxicity and inflammation than commonly used adhesive CA-Lp. Multifunctional X-ray fluorescence and CT properties [196].

The effect of high *Z* and *ρ* on X-ray attenuation can be expressed by the following relation [190]:

$$µ=\frac{\rho Z^{4}}{AE^{3}}$$

where µ is the X-ray attenuation coefficient, *ρ* the density, *Z* the atomic number, *A* the atomic mass and *E* the X-ray energy. 

In clinical CT, X-ray attenuation is measured in Hounsfield units (HUs). The value of HUs can be determined from the corresponding value of µ by the following formula [190,197]:

$$\mathrm{HU}=\frac{\left(µ-{µ}_{water}\right)}{\left({µ}_{water}-{µ}_{air}\right)}\times 100$$

CT devices are calibrated with respect to water; on this scale, the radiodensity of water is taken as 0 HU, whereas air is 1000 HU. The value of HU for bone may be about +1000 and for soft tissues, such as protein, it may vary between −100 to +100 HU. For the visualisation of the tissue of interest, a minimum difference of about 50–100 HU is requisite; however, the greater the difference, the better the visualisation [198]. 

## 12. Bismuth Oxide and Hydroxide Nanostructures

Bismuth (Bi)-containing NPs have recently attracted much interest due to their multifunctional and theranostic properties. Bismuth has a high atomic number (Z = 83), which can offer excellent X-ray attenuation (5.74 cm^2^ kg^−1^ at 100 keV) [199,200]. Owing to the range of Bi properties, such as high surface area, chemical inertness, low toxicity, diamagnetism, antibacterial activity, catalytic performance, strong near-infrared absorbance and high efficiency of photothermal conversion [201,202], it can be employed as a multifunctional and theranostic agent. The use of Bi compounds in conventional medicines dates back to the 18th century [203]. It has been employed in various pharmaceutical products to treat gastrointestinal disorders, syphilis and hypertension. Nowadays, the use of Bi-based NPs has been expanded to advance diagnostic and therapeutic applications, such as X-ray radiotherapy, biosensing, bioimaging, cancer therapy, photothermal therapy, antimicrobial formulations, ROS-based therapy and tissue engineering [202,204]. A variety of Bi-based nanostructures, such as Bi NPs, bismuth oxide (Bi_2_O_3_) NPs, Bi(OH)_3_ NPs, bismuth sulphide (Bi_2_S_3_), bismuth selenide (Bi_2_Se_2_), bismuth ferrite (BiFeO_3_), bismuth tungstate (Bi_2_WO_6_) and bismuth dimercaptopropanol (BisBAL), can be synthesised using a suitable synthesis method. Synthesis methods, such as hydrothermal processing, chemical precipitation, sol–gel technique, thermal evaporation, micro-emulsion, sonochemical synthesis, solvothermal synthesis and microwave irradiation, can be selectively employed for the desired size and shape of NPs.

**Figure 19:**
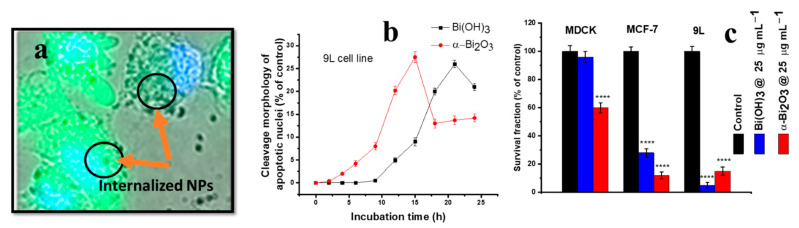
(**a**) Overlay image of double-stained (Hoechest and H_2_DCFDA) 9L cells; (**b**) apoptotic cells with cleavage morphology of 9L cells after treatment with NPs (50 µg mL^−1^) for 0, 2, 4, 6, 9, 12, 15 and 24 h compared to the control; (**c**) clonogenic assay of MDCK, MCF-7 and 9L cell lines after treatment with Bi(OH)_3_ and α-Bi_2_O_3_ NPs (25 µg mL^−1^) for 24 h. Where **** *p* ˂ 0.0001, *** *p* ˂ 0.001, ** *p* < 0.01 and * *p* < 0.05 vs. control. Images and graphs are reproduced with permission from [205], copyright 2018, Elsevier, and represent the overall trend of results.

The use of Bi-based nanostructures is rapidly expanding in the field of cancer diagnosis and treatment as they have shown promising results. Stewart et al. synthesised Bi_2_O_3_ NPs by the precipitation method and reported the selective ROS-dependent toxicity in malignant (9L cell line) and non-malignant (MDCK cell line) cells [204]. Bogus et al. reported biocompatible Bi(OH)_3_ with low photocatalytic activity and good UV absorption properties [205]. In the present study, Bi(OH)_3_ exhibited low toxicity towards MDCK cells, which resulted in higher cell viability (~83%) at a tested dose of 25 µg mL^−1^ over a period of 24 h (Figure 19c), whereas higher toxicity was observed in MCF-7 and 9L (with survival fraction ~20 and 5%) cells. The compared data of Bi_2_O_3_ NP treatment also showed similar trends of toxicity in malignant (MCF-7 and 9L) cells; however, the cell survival fraction lowered to 60% in treated MDCK cells at a 25 µg mL^−1^ NP concentration. The confocal microscopy of strained (Hoechest and H_2_DCFDA) 9L cells showed the cellular internalisation of Bi(OH)_3_ NPs (Figure 19a). The treatment of 9L cells with Bi(OH)_3_ and Bi_2_O_3_ NPs resulted in the highest cellular apoptosis after 24 and 15 h, respectively, as shown in Figure 19b.

Recently, Yang et al. developed lipid (1,2-dilauroyl-sn-glycero-3-phosphocholine, abbreviated as DLPC)-coated Bi NPs for efficient photothermal therapy, CT imaging and tumour irradiation by NIR light irradiation [201]. Bi@DLPC NPs showed a higher cellular uptake and tumour accumulation via the EPR effect, and changed cell membrane permeability and caused mitochondrial dysfunction. A variety of Bi-based nanostructures have been prepared for theranostic applications using different synthesis techniques, including NPs, coated NPs, functionalised NPs and compounds of Bi with different elements. A brief summary of the synthesis method, size, shape and in vitro behaviour of different reported structures is presented in Table 8. Recently, Shahbazi et al. published a comprehensive review on the multifunctional application of Bi-based nano- and composite structures, and also highlighted the synthesis methods used for the development or functionalisation of Bi-based NPs [202].

## 13. Magnesium Oxide Nanostructure

Currently, research is being conducted on therapeutic aspects, such as the antibacterial, cytotoxic and antithrombotic properties of MgO structures. MgO has also been used in nano-cryosurgery for the treatment of cancer [214,215]. In addition to the research on the therapeutic aspects of MgO nanostructures, the focus has also been shifted to the imaging capabilities of MgO. Extensive point defects in the MgO structure have been studied to exploit the maximum photoluminescence effect. Near-infrared signals from MgO crystals excited by 325 nm and 532 nm radiation for the broad fluorescence band have been studied by Prucnal et al. [216]. This study aligned the 800 nm broad line to Cr^+3^ substitutional defects as excited by a 532 nm laser, and the line above 850 nm assigned to V^+2^ substitutional defects. The lines above 850 nm are strongly temperature dependent with a maximum intensity at 15 K, and linked to a phonon-assisted transition [216]. Kunz et al. reported the emission at 700 nm in MgO crystals by excitation at 5–7 K, which is linked to Cr^+3^ defects [217]. The fluorescence properties help to track the interaction of MgO NPs inside cells and other biological entities. In addition to intrinsic fluorescence, MgO NPs also have good biocompatibility and biodegradability. The combination of biocompatibility, therapeutic effect and imaging capabilities make MgO NPs a theranostic agent for biological applications. MgO NPs can be employed in cutting-edge treatment by fluorescence-guided surgery. These theranostic NPs can help to differentiate between the normal surrounding tissues from tumour tissues through fluorescence-guided therapy. Furthermore, controlling the size and defect concentrations of nanostructures can enhance the permeability and retention of NPs or increase the capability of binding selectivity towards cancer cell receptors for recognition. Khalid et al. reported bright-red florescence in HaCaT, fibroblast cells (3T3) and prostate cancer (PC-3) cells by employing commercial (spherical, 20–40 nm) and ball-milled (irregular shape, 70–230 nm) MgO NPs, Table 9.

Due to the alkaline nature of MgO powder, O_2_^-^ can form a concentrated layer on the surface. When MgO NPs interact with bacteria and other cells, O_2_^−^ reacts with the H^+^ ion to form HO^•^, which increases the antibacterial activity. The release of Mg^+2^ also increases antibacterial activity by ROS generation and can cause cellular enzyme deactivation and mitochondrial impairment. The generation of excessive ROS can cause oxidative stress and damage to the membrane and other cellular components, hence leading to cell necrosis (Table 9) [222].

$$O_{2}^{-}+{\;H}^{+}\to {\;\mathrm{OH}}^{•}$$

**Figure 20:**
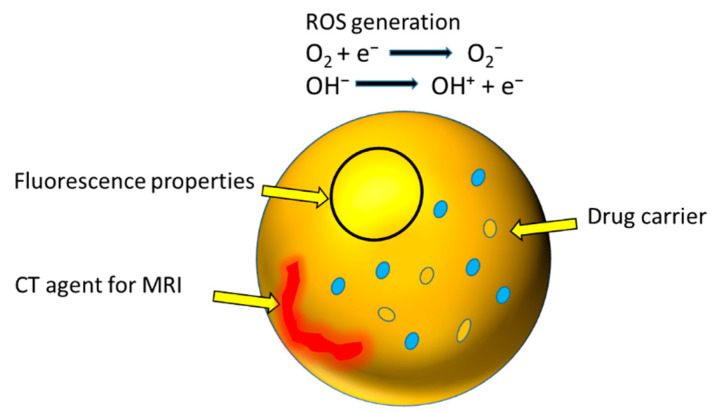
ROS generation by multifunctional MgO nanocrystals for cancer treatment and diagnosis.

In short, MgO NPs can increase the concentration of ROS (Figure 20), decrease the level of glutathione and increase lipid peroxidation in tumour cells [223]. Krishnamoorthy et al. reported a strong interaction between alkaline MgO NPs and acidic cancer cells [221], in addition to the MRI contrast properties, hyperthermia ability and nano-cryosurgery applications in the treatment of cancer also evaluated by Krishnamoorthy et al. [221]. They observed higher toxicity in malignant cell lines (SNU-A6, AGS) and recorded non-significant toxicity in a non-malignant human lung fibroblast (CCD-25Lu) cell line. This study confirmed the generation of ROS by MgO NPs that caused lipid peroxidation and oxidative stress-mediated cell death [221]. It has also been reported that MgO NPs are non-toxic and biocompatible towards many human cell lines under 300 µg mL^−1^, which has been published in a study conducted by Mahmoud et al. [218]. They can be coated with antitumour drugs, such as albumin, doxorubicin and 2-Metoxyestradiol, to construct a more effective anticancer theranostic system [220]. Biocompatible and safe MgO NPs can be developed by green synthesis or functionalisation with non-toxic materials. These NPs can be an excellent choice for controlled therapy and the diagnosis of cancer.

**Figure 21:**
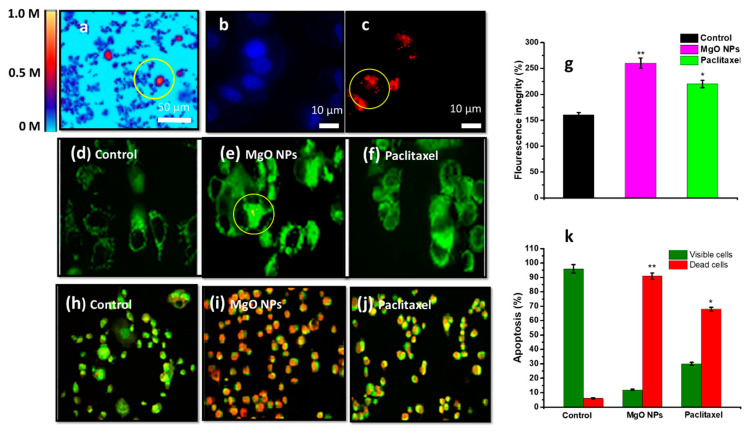
(**a**) Two-dimensional (2D) fluorescence maps (200 × 200 µm^2^) of MgO NPs; (**b**,**c**) wide-field fluorescence images of breast cancer cells (MCF-7) treated with MgO NPs for 24 h and taken at excitation wavelengths of 390 nm and 560 nm, respectively; (**d**–**f**) fluorescence images of MCF-7 cells treated with 123 MgO NPs for intracellular ROS observation with reference to the control and paclitaxel; (**g**) bar chart of quantification of ROS from fluorescence maps; (**h**–**j**) antiproliferative effect caused by MgO NP treatment visualised using double staining (AO and EtBr dyes) at IC_50_ in MCF-7 cells. Where ** *p* < 0.01 and * *p* < 0.05 and are considered significant. Images (**a**–**c**) are reproduced with permission from [224], copyright 2021, Nanomaterials (MDPI), whereas images (**d**–**f**,**h**–**j**) and charts (**g**,**k**) are reproduced with permission from [225], copyright 2020, PLoS ONE.

The fluorescence properties of MgO NPs studied by Li et al. using two-dimensional confocal maps at a 532 nm excitation with a green laser are shown in Figure 21a [224]. The scale bar on the right side of Figure 21a represents the counts (0–1 M counts per second). The encircled bright regions show the MgO NPs fluorescence when scanned at a low excitation power (80 µW). Wide-field fluorescence images of MgO NP-treated MCF-7 cells at two different excitations (390 nm and 560 nm) indicate the green and red fluorescence, respectively (Figure 21b,c) [224]. In another study, Amina et al. used MgO NPs as an anticancer agent. This study reported an increased amount of intracellular ROS in MgO NP-treated cells compared to control and chemotherapeutic drugs (paclitaxel), as shown in Figure 21d, e and f. The enhanced green fluorescence in the treated cells exhibited ROS formation, which caused an apoptotic effect in MCF-7 cells. The ROS-related fluorescence integrity of cells was quantified in the form of a bar graph, as shown in Figure 21g. A higher ROS formation in MgO NP-treated cells caused higher cellular apoptosis and, hence, higher fluorescence [224]. Furthermore, dual staining using acridine orange (AO) and ethidium bromide (EtBr) dyes was performed to assess the apoptosis induced by MgO NPs. The presence of green fluorescence caused the penetration of AO dye into plasma and the binding of the DNA of cells, where the green colour represents the viable cells. On the other hand, orange-red fluorescence was caused due to the penetration of EtBr through damaged cell membranes, indicating apoptotic and dead cells (Figure 21h,i and j). The quantification of the obtained results demonstrates an increased dead cell percentage relative to the viable cells (84.3 ± 0.01% dead and 15.7 ± 0.02% viable cells) in MgO NP-treated cells compared to the control (8.3 ± 0.07% dead and 91.8 ± 0.09% viable cells) and paclitaxel-treated (65.4 ± 0.06% dead and 34.6 ± 0.04% viable cells) groups, as shown in Figure 21k. The treatment of MCF-7 cells with MgO NPs resulted in the production of ROS, which caused oxidative stress-mediated cell death. Additionally, MgO NPs disrupted the membrane mitochondrial potential (MMP) and led to cell death through intrinsic pathways of apoptosis [224].

## 14. Elimination of Nanoparticles from the Body after Treatment

The elimination of nanoparticles from the body after their intended use is required in order to avoid long-term toxicity and other health complications. Currently, there are different elimination pathways that highly depend upon the size, shape and biodegradability of nanoparticles. Most nanoparticles are resistant to metabolism and renal secretion because of their large size [226]. Intravenously administered nanoparticles can be eliminated by two main pathways: (1) renal elimination and (2) hepatobiliary elimination [227]. Choi et al. conducted a renal excretion experiment using nanoparticles with different hydrodynamic diameters and showed that quantum dots with a diameter < 5.5 nm exhibited efficient renal excretion due to the limitation of the pore size of glomerular filtration. Various studies have been performed to optimise the renal elimination of nanoparticles by the fine tuning of size, shape and surface charge. 

Gold nanoparticles are FDA-approved metallic nanoparticles that are employed for various biological roles. Their elimination mechanism is also crucial after treatment, according to various studies. 

Au nanoparticles with a hydrodynamic diameter < 5.5 nm are easily secreted by the renal rout; however, larger Au nanoparticles can accumulate in the spleen and liver and follow the reticuloendothelial rout for elimination [228,229]. W. Poon et al. reported the hepatobiliary elimination of non-biodegradable Au nanoparticles and found that Kupffer and sinusoidal endothelial cells are major abstractions in the elimination of nanoparticles. These nonparenchymal cells have a high phagocyte affinity for NPs and most NPs are trapped by these cells upon their first interaction. The elimination through this pathway requires further study for understanding NPs’ interaction with biological components, such as the bile duct, hepatocytes and intestines [227].

The elimination of silver-based NPs is also challenging, Zande et al. reported silver NPs’ uptake and elimination in rat organs. In this study, silver NPs were cleared from most rat organs, except the brain and testis [230]. Similarly, other inorganic NPs are being investigated for complete removal from the body in a specific time limit. Inorganic NPs are largely sequestered from the blood by liver and spleen, and thereby increase the chances of accumulation. A sever uptake of inorganic NPs can be avoided by poly(ethylene glycol) coating, but it does not guarantee complete elimination [231,232]. The size, shape and surface charge affect the renal clearance of inorganic nanoparticles from the body. Inorganic NPs are usually developed with controlled properties as an efficient option for early diagnosis and therapy. These properties include: theranostic characteristics, high clearance rate, good physiological stability, low binding affinity for biomolecules, low accumulation, high specific target capability, short distribution life and high permeability and retention [231]. Currently, various studies are being conducted to develop composite core–shell structures with shells consisting of biodegradable molecules and cores having inorganic quantum-sized particles (<6 nm) [231,233,234].

## 15. Conclusions

The physio-chemical properties of nanomaterials play a critical role in their cytotoxicity and multifunctional role. Au, Ag-based nanostructures have a very effective role in malignant tumour treatment due to their antiviral and anti-infective properties, but, at the same time, they produced toxicity in normal cells, such as HaCaT, human foetal osteoblasts and human HepG2 cells. Similarly, the oxide nanostructures of Mn and Fe increased oxidative stress through ROS generation, which is a critical factor for oxidative bursts in cancer cells. However, they also caused toxicity in normal cells, such as CCL-149, L929, BEAS-2B and HepG2. Increased ROS production and nanotoxicity decreased cell proliferation and viability. The damage of cell membrane, mitochondrion, DNA and depletion of necessary enzymes and proteins led to cell death, which are desired in tumour cells. In a few studies, it has been reported that the NPs of Au, Ag and Mn also caused genotoxicity and neurotoxicity. 

The toxic behaviour of some metal/metal oxide NPs systems, particularly, naked NPs towards normal cells, is a matter of concern. Many biocompatible systems with selective toxicity in the microenvironment have been synthesised using controlled processing parameters, such as a suitable chemical selection and synthesis environment. 

Recently, developed superparamagnetic nanostructures of γ-Fe_2_O_3_ showed higher toxicity in MDA-MB-231 and MCF-7 cells compared to HaCaT cells. A confocal observation also showed their fluorescence imaging capability. Oxygen-deficient La_2_O_3_ synthesised by spray pyrolysis and the spray precipitation method exhibited higher UV absorption and ROS scavenging in HaCaT cells. Similarly, ITO nanoparticles that were synthesised under a controlled atmosphere resulted in a high level of ROS in MDA-MB-231 and MCF-7 cell lines compared to the MCF-10A cell line. The selective generation of ROS and fluorescence imaging features of ITO NPs presented them as suitable candidates for cancer theranostics. Ta_2_O_5_ NPs were successfully employed in radiation dose enhancement and fluorescence imaging. They also showed a good drug loading capability. Enhanced biocompatibility and increased UV absorption was witnessed in the case of CeO_2_ nanodot-encrusted TiO_2_ nanostructures. Bi-based nanostructures showed excellent theranostic behaviour in malignant tumour (9L and MCF-7) cells. An increased level of ROS caused an antiproliferative effect in MCF-7 cells and excellent traceability when treated with MgO NPs. Nanostructures exhibiting size- and shape-dependent theranostic properties, such as star-, cube- and rod-shaped nanoparticles, caused higher toxicity than spherical-shaped nanostructures. Similarly, nano-sized particles in the 10–30 nm range offered excellent theranostic properties, and even quantum-sized particles showed excellent tumour penetration, drug loading and diagnostic capabilities.

The imaging (such as fluorescence, MRI and CT) and therapeutic capabilities (such as hyperthermia, photothermal, ROS generation and ultrasonic therapy) of efficient nanosystems were exhibited in the tumour microenvironment. The effect on normal cells during the treatment was highlighted. In summary, metal and metal oxide nanosytems have proven to be efficient in selective tumour diagnosis and treatment. The reported toxicity towards non-malignant cells in a few cases can be minimised through different approaches, such as encrustation, encapsulation, polymer coatings, core–shell structures, NP surface binding and inert atmosphere annealing to increase the biocompatibility and effectiveness of disease treatment of theranostic nanosystems. The presented summary of the critical features of nanostructures will serve as a fundamental tool to analyse, select and modify the properties of existing nanosystems in order to develop an efficient drug/system that selectively generates oxidative stress in tumour cells. The developed nanostructures may generate selective toxicity due to controlled ROS generation alongside diagnostic properties, and hence can offer safe, efficient and economic treatment options. Furthermore, a comprehensive study on the reported individual nanotheranostic systems (coated and uncoated) covering in vivo performance will be conducted to facilitate the easy selection of nanosystems for a particular use or research.

## Figures and Tables

**Table 6:** ITO NPs’ cytotoxicity in malignant and non-malignant cells reported by Hsu et al. [157].

Cell Line	NP Size and Shape	Synthesis Method	Effect
Human breast cancer (MDA-MB 231) cell line	ITO NPs, irregular (10.78 nm)	Chemical precipitation and calcination	Higher NP uptake, increased ROS generation caused higher oxidative stress, cellular apoptosis evaluated by Annexin-V binding [157].
Breast cancer (MCF-7) cell line	ITO NPs, irregular (10.78 nm)	Chemical precipitation and calcination	ROS induced oxidative stress, cellular apoptosis [157].
Human epithelial (MCF-10A) normal breast cells	ITO NPs, irregular (10.78 nm)	Chemical precipitation and calcination	ROS scavenging, lower cellular apoptosis due to lower oxidative stress [157].

**Table 8:** Size, shape, and synthesis methods of Bi-based NPs and their in vitro cellular behaviour.

Cell Line	NP Size and Shape	Synthesis Method	Effect
9L cell line, MCF cell line	α-Bi_2_O_3_ (6–10 nm, round and ellipsoidal shapes)	Precipitation method	Higher cellular internalisation, increased ROS generation, induced higher toxicity and, hence, lowered cell viability [205].
MDCK cell line	α-Bi_2_O_3_ (6–10 nm, round and ellipsoidal shapes)	Precipitation method	Lower cellular internalisation, decreased ROS generation and, hence, minimum toxicity generation, biocompatible behaviour [205].
HeLa cells	Bi_2_S_3_ (2–3 nm, nanodots, coated with PVP)	Hot injection method	Dose-dependent uptake, good biocompatibility even at higher doze (2 mg Bi mL^−1^, excellent for CT imaging [206].
KB cells, A549 cell lines	Bi_2_O_3_ (15–25 nm, hexagonal shape)	Solvothermal synthesis	Higher ROS generation and toxicity of bare Bi_2_O_3_ NPs than folic acid-coated NPs, significant increase in oxidative stress by bare Bi_2_O_3_ NPs [207].
HT-29 cell line	Bi_2_O_3_ (40–120 nm, spherical shape)	Biogenic synthesis	Reduced GSH level, increased MDA level, decreased SOD and CAT activities, induction of cytotoxicity through late apoptosis [208].
Mice lung epithelial and fibroblast cells	Bi_2_Se_3_ (70 nm, flake-like shape)	Sonochemical method	Cytotoxic inflammation due to increased expression of IL-1β, MIP-2 and IL-6; increased oxidative stress through neutrophilic and ROS generation [209].
HeLa cells	Bi_2_S_3_ decorated with chitosan and RGD peptide (nanosheets, 53.8 nm wide and 6 nm thick)	Solution-based method using poly(vinylpyrrolidone)	Mitochondria-mediated intrinsic cell apoptosis, G0/GI cell cycle arrest, TrxR inhibition, ROS generation, X-rays induced apoptosis, increased radio-sensitisation effect. Excellent photoacoustic imaging [210].
Murine macrophage cell line (RAW 264.7)	Bi NPs (unknown 25–60 nm)	Laser ablation method	ROS generation and increased oxidative stress, DNA and plasma membrane damage, increased phagocytic activity [211].
MCF-7 cell line	Bi_2_O_3_ (unknown 14 nm)	Commercial	Increased ROS generation, SOD and CAT activity, and increased GSH concentration, mitochondrion dysfunction, DNA damage and cellular apoptosis [212].
HaCaT and MDCK cell lines	Bi(OH)_3_ (6 nm, spherical shape)	Precipitation method	Higher cell viability, biocompatible nature of the synthesised NPs. No DNA or mitochondrion damage [213].

**Table 9:** Size, shape, and synthesis of MgO NPs and their in vitro behaviour in different cell cultures.

Cell Line	NP Size and Shape	Synthesis Method	Effect
Prostate cancer (PC-3) cells	Spherical, commercial (20–40 nm), irregular shape, ball milling (70–230 nm)	Commercial (MTI Corporation, St. Richmond, CA, USA), ball milling	Dose-dependent toxicity at concentration of ≥62.5 µg mL^−1^. Fluorescent imaging properties [217].
Human liver cancer (HepG2) cell lines	Unknown (20 nm)	Thermal decomposition of metal–oleate complex	No significant toxicity towards oxidative stress genes (GST and catalase), demonstrated high biocompatibility and low toxicity, also recorded high stability in microenvironment [215].
Human lung (A549) cells	Irregular shape (43.9 nm)	Commercial (Sigma-Aldrich, St. Louis, Missouri, USA)	Generation of ROS, oxidative damage and depletion of GSH, mitochondrial apoptosis and DNA damage [218].
Human colon adenocarcinoma (HT-29)	Irregular shape (50 nm)	Precipitation–aging–calcination method	Lipid peroxidation, increased ROS generation oxidative stress, depletion of GSH, overall dose-dependent toxicity [219].
Prostate cancer cell line (LNCap)	Spherical (15–30 nm), PEG-coated (93 nm)	Sol–gel method	Decrease in cell viability, coated MgO NPs used as anticancer drug carriers [220].
(HeLa), human gastric adenocarcinoma and carcinoma (AGS, SNU-A6) cell line	Irregular shape (20 nm)	Precipitation method	Ultrasound induced lipid peroxidation, increased ROS level, ROS induced cellular apoptosis [221].

## Data Availability

The study did not report any data.
